# Photodissociation of particulate nitrate as a source of daytime tropospheric Cl_2_

**DOI:** 10.1038/s41467-022-28383-9

**Published:** 2022-02-17

**Authors:** Xiang Peng, Tao Wang, Weihao Wang, A. R. Ravishankara, Christian George, Men Xia, Min Cai, Qinyi Li, Christian Mark Salvador, Chiho Lau, Xiaopu Lyu, Chun Nan Poon, Abdelwahid Mellouki, Yujing Mu, Mattias Hallquist, Alfonso Saiz-Lopez, Hai Guo, Hartmut Herrmann, Chuan Yu, Jianing Dai, Yanan Wang, Xinke Wang, Alfred Yu, Kenneth Leung, Shuncheng Lee, Jianmin Chen

**Affiliations:** 1 Department of Civil and Environmental Engineering, the Hong Kong Polytechnic University, Hong Kong, 999077 China; 2 Department of Ambient Air Quality Monitoring, China National Environmental Monitoring Center, Beijing, 100012 China; 3 Hangzhou PuYu Technology Development Co., Ltd, Hangzhou, Zhejiang 311300 China; 4 Departments of Atmospheric Science and Chemistry, Colorado State University, Fort Collins, CO 80523 USA; 5 Univ Lyon, Université Claude Bernard Lyon 1, CNRS, IRCELYON, Villeurbanne, 69626 France; 6 Institut de Combustion, Aérothermique, Réactivité et Environnement (ICARE), CNRS/OSUC, 45071 Orléans, Cedex 2 France; 7 Department of Atmospheric Chemistry and Climate, Institute of Physical Chemistry Rocasolano, CSIC, Madrid, 28006 Spain; 8 Department of Chemistry and Molecular Biology, University of Gothenburg, Gothenburg, 40530 Sweden; 9 Air Science Group Environmental Protection Department, HKSAR, Hong Kong, 999077 China; 10 Research Center for Eco-Environmental Sciences, Chinese Academy of Sciences, Beijing, 100085 China; 11 Leibniz Institute for Tropospheric Research (TROPOS), Atmospheric Chemistry Department (ACD), 04318 Leipzig, Germany; 12 School of Environmental Science and Engineering, Shandong University, Qingdao, Shandong 266237 China; 13 Environment Research Institute, Shandong University, Qingdao, Shandong 266237 China; 14 Department of Environmental Science and Engineering, Fudan University, Institute of Atmospheric Sciences, Shanghai, 200433 China; 15 Present Address: Balik Scientist Program, Department of Science and Technology - Philippine Council for Industry, Energy and Emerging Technology Research and Development, Bicutan, Taguig, 1630 Philippines; 16 Present Address: Environmental Modeling Group, Max Planck Institute for Meteorology, Hamburg, 20146 Germany

**Keywords:** Atmospheric chemistry, Atmospheric chemistry

## Abstract

Chlorine atoms (Cl) are highly reactive and can strongly influence the abundances of climate and air quality-relevant trace gases. Despite extensive research on molecular chlorine (Cl_2_), a Cl precursor, in the polar atmosphere, its sources in other regions are still poorly understood. Here we report the daytime Cl_2_ concentrations of up to 1 ppbv observed in a coastal area of Hong Kong, revealing a large daytime source of Cl_2_ (2.7 pptv s^−1^ at noon). Field and laboratory experiments indicate that photodissociation of particulate nitrate by sunlight under acidic conditions (pH < 3.0) can activate chloride and account for the observed daytime Cl_2_ production. The high Cl_2_ concentrations significantly increased atmospheric oxidation. Given the ubiquitous existence of chloride, nitrate, and acidic aerosols, we propose that nitrate photolysis is a significant daytime chlorine source globally. This so far unaccounted for source of chlorine can have substantial impacts on atmospheric chemistry.

## Abstract (web summary)

This study unravels an important daytime Cl_2_ source in the extra-polar atmosphere and shows that photolysis of particle nitrate at high acidity produced unprecedented levels of Cl_2_, boosting the oxidative power and air pollutants like O_3_.

## Introduction

Atomic chlorine (Cl) is a very reactive radical, known to destroy stratospheric ozone (O_3_) through catalytic cycles^1,2^. In the lower troposphere, it can initiate the oxidation of volatile organic compounds (VOCs), increase the levels of conventional radicals (OH, HO_2_ and RO_2_), and produce O_3_ and secondary organic aerosols (SOA)^3–7^ which are air pollutants and also alter the Earth’s radiation budget and climate. Cl reacts rapidly with methane, the most abundant hydrocarbon and the second-most important greenhouse gas emitted into the atmosphere^8,9^. Molecular chlorine (Cl_2_) is an important Cl precursor. It can be photolyzed quickly to release two Cl atoms during the daytime, and its production through heterogeneous reactions is a key step in the O_3_ destruction over Antarctica during austral spring^10^. Previously, Cl_2_ has been measured in the lower troposphere in locations such as at the Arctic surface^11,12^, the marine boundary layer^13–15^, and continental sites^16,17^. Cl_2_ was found to typically peak during nighttime, but elevated levels (17–450 pptv) have also been observed during daytime^6,11,12,18–21^. The daytime occurrence of Cl_2_ is of great importance as it may have a profound impact on atmospheric photochemistry and oxidation capacity^6,19^. Such observations also reveal the existence of a significant Cl_2_ source that compensates or even overcomes its fast photolytic loss. Although daytime Cl_2_ can be emitted from various sources, such as from coal combustion^16^ or water treatment facilities^15^, it can also be produced through some photochemical processes^11,18–20^. However, the underlying photochemical mechanisms remain uncertain. As a result, current state-of-the-art air quality models do not typically implement such chemistry, and therefore, cannot reproduce the observed high daytime Cl_2_ levels in polluted regions^7,22^. Consequently, the impact of Cl_2_ on atmospheric oxidation is currently underestimated. Finally, as there were just only handfuls of Cl_2_ observations outside the polar regions to date^13–17,19–21^, our ability to assess the Cl_2_ and Cl impact in different parts of the world is still very limited.

In this work, we report atmospheric observations of Cl_2_ and other chemicals obtained at a polluted coastal site in southern China during autumn 2018. The Cl_2_ concentrations are much higher than those previously measured outside polar regions. We show that previously proposed Cl_2_ production mechanisms cannot account for the large Cl_2_ daytime source at this site and this source positively correlates with solar radiation, particulate nitrate, and particulate surface area. Laboratory experiments show that illuminating solution of sodium chloride and nitrate under acidic condition and ambient particulates can produce a large amount of Cl_2_, which can explain a large fraction of the observed Cl_2_ at our site. We propose that nitrate photolysis at high aerosol acidity is an important pathway for activating inert chloride to produce photoliable Cl_2_ during daytime in the polluted troposphere. Model calculations demonstrate significant enhancement of conventional radical levels, hydrocarbon oxidation, and ozone production by the high levels of Cl_2_ at the study site. We suggest that the same Cl_2_ production pathway may exist in other places of the world and call for more attention to the role of Cl_2_ in tropospheric chemistry and air quality of polluted regions.

## Results and Discussion

### Field observations

**Fig. 1:**
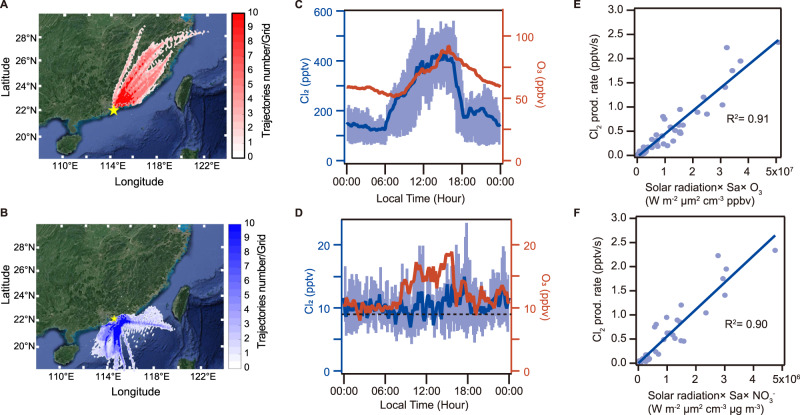
Ambient observations at Cape D’ Aguilar, Hong Kong, from 31 August to 9 October 2018. Back trajectories of air mass from (**A**) continental region (5 September - 9 October) and (**B**) the South China Sea (31 August - 4 September). Contour represents the number of trajectories in each 0.1-degree latitude × 0.1-degree longitude grid. The diurnal profiles of Cl_2_ and O_3_ (**C**) in the air mass from the continental region (5 September - 9 October); (**D**) in the air mass from the South China Sea (31 August - 4 September). The dashed line represents the detection limit of the CIMS instrument. The blue line is the 10-min average of Cl_2_, and the blue shade represents the 25 percentile and 75 percentile values. The red line is the 10-min average of O_3_. (**E**) The scatter plot of the production rate of Cl_2_ (${P}_{{{Cl}}_{2}}$) and the product of the solar actinic flux (W m^−2^), the aerosol surface area density (Sa, μm^2^ cm^−^^3^), and O_3_ mixing ratio (ppbv) from 08:00 to 18:00 in the continental air mass. (**F**) The scatter plot of the production rate of Cl_2_ (${P}_{{{Cl}}_{2}}$) and the product of the solar actinic flux, the aerosol surface area density (Sa, μm^2^ cm^−3^), and nitrate concentration in PM_10_ (μg m^−3^) from 08:00 to 18:00 in the continental air mass. The ${P}_{{{Cl}}_{2}}$equals the photolysis rate of Cl_2_ (${J}_{{{Cl}}_{2}}$× measured Cl_2_ concentration) as Cl_2_ was near a photo stationary state. ${J}_{{{Cl}}_{2}}$ was calculated from the TUV model (http://cprm.acom.ucar.edu/Models/TUV/Interactive_TUV) under clear sky conditions and then scaled to the solar radiation derived J_NO2_ (see Methods section “Chemical box model”).

To investigate the abundance, sources, and impact of Cl_2_, we measured its concentrations using a chemical ionization mass spectrometer (CIMS) (Methods section “Field measurements”) at a coastal site in Hong Kong (Cape D’Aguilar, 22.21°N, 114.25°E, Supplementary Fig. 1), adjacent to the highly industrialized Pearl River Delta (PRD). The field measurement took place from 31 August to 9 October 2018, when this site predominantly received outflow of air from eastern and southern China and occasionally inflow of marine air and spillover of urban pollution from Hong Kong (HK) and other PRD cities^23,24^ (Fig. 1A, B, also see Methods section “HYSPLIT and E-AIM models”). Moderate to very high mixing ratios of ozone (up to 186 ppbv) (Supplementary Fig. 2) were observed during the study, indicating active photochemistry during the measurement period.

We frequently observed Cl_2_ mixing ratios greater than 400 pptv (10-min average) with a maximum of 998 pptv (Supplementary Fig. 2), which is much higher than the values reported in the limited Cl_2_ measurements^6,9,19^. The Cl_2_ mixing ratio exhibited a distinct daytime peak (Fig. 1C and Supplementary Fig. 2), coinciding with that of ozone. Much higher Cl_2_ levels were observed in the air mass originating from inland than that from the ocean, indicating the important role of anthropogenic pollution in producing the observed high Cl_2_ (Fig. 1C, D). The highest Cl_2_ (and O_3_) occurred on 11 September 2018 in a heavy photochemical pollution episode (Supplementary Fig. 2), when the site was impacted by plumes from HK and other PRD cities^25^. With an average photolysis lifetime of Cl_2_ of about 7 min at noon during this study, sustained high levels of daytime Cl_2_ must arise from a significant in-situ production, with an average production rate of up to 2.7 pptv s^−1^ at noon. ClNO_2_—another Cl precursor—exhibited typical nighttime peaks with the highest mixing ratio of 1900 pptv, comparable to the value observed in our previous measurements at a nearby site^26^.

Previous studies have proposed two chemical mechanisms to explain the observed daytime Cl_2_ production. The first one involves the aqueous-phase reaction of OH with chloride in the solution or at the air-water interface, with OH being produced from O_3_ photolysis in the gas or aqueous phase (R1–R9)^27,28^.

R1 $${{{{{{\rm{O}}}}}}}_{3({{{{{\rm{g}}}}}})}+{{{{{\rm{h}}}}}}{\upnu} \to {{{{{\rm{O}}}}}}({\!\,}^{1}{{{{{\rm{D}}}}}})_{({{{{{\rm{g}}}}}})}+{{{{{\rm{O}}}}}}_{2({{{{{\rm{g}}}}}})}$$

R2 $${{{{{\rm{O}}}}}}{({\!\,}^{1}{{{{{\rm{D}}}}}})}_{({{{{{\rm{g}}}}}})}+{{{{{{\rm{H}}}}}}}_{2}{{{{{\rm{O}}}}}} \to {{{{{{\rm{2OH}}}}}}}_{({{{{{\rm{g}}}}}})}$$

R3 $${{{{{{\rm{OH}}}}}}}_{({{{{{\rm{g}}}}}})}+{{{{{{\rm{Cl}}}}}}}_{({{{{{\rm{interface}}}}}})}^{-} \to {({{{{{\rm{OH}}}}}}\ldots {{{{{{\rm{Cl}}}}}}}^{-})}_{({{{{{\rm{interface}}}}}})} \to 1/2\,{{{{{{\rm{Cl}}}}}}}_{2({{{{{\rm{g}}}}}})}+{{{{{{\rm{OH}}}}}}}_{({{{{{\rm{aq}}}}}})}^{-}$$

R4 $${{{{{{\rm{OH}}}}}}}_{({{{{{\rm{aq}}}}}})}+{{{{{{\rm{Cl}}}}}}}_{({{{{{\rm{aq}}}}}})}^{-}\rightleftharpoons {{{{{{\rm{HOCl}}}}}}}_{({{{{{\rm{aq}}}}}})}^{-}$$

R5 $${{{{{{\rm{HOCl}}}}}}}_{({{{{{\rm{aq}}}}}}\,{{{{{\rm{or}}}}}}\,{{{{{\rm{interface}}}}}})}^{-}+{{{{{{\rm{H}}}}}}}_{({{{{{\rm{aq}}}}}})}^{+}\rightleftharpoons {{{{{{\rm{HClOH}}}}}}}_{({{{{{\rm{aq}}}}}})}$$

R6 $${{{{{{\rm{HClOH}}}}}}}_{({{{{{\rm{aq}}}}}})}\rightleftharpoons {{{{{{\rm{Cl}}}}}}}_{({{{{{\rm{aq}}}}}})}+{{{{{{\rm{H}}}}}}}_{2}{{{{{\rm{O}}}}}}$$

R7 $${{{{{{\rm{Cl}}}}}}}_{({{{{{\rm{aq}}}}}})}+{{{{{{\rm{Cl}}}}}}}_{({{{{{\rm{aq}}}}}})}^{-}\to {{{{{{\rm{Cl}}}}}}}_{2({{{{{\rm{aq}}}}}})}^{-}+{{{{{{\rm{H}}}}}}}_{2}{{{{{\rm{O}}}}}}$$

R8 $${{{{{{\rm{2Cl}}}}}}}_{2({{{{{\rm{aq}}}}}})}^{-}\to {{{{{{\rm{Cl}}}}}}}_{2({{{{{\rm{aq}}}}}})}+{{{{{{\rm{2Cl}}}}}}}_{({{{{{\rm{aq}}}}}})}^{-}$$

R9 $${{{{{{\rm{Cl}}}}}}}_{2({{{{{\rm{aq}}}}}})}\to {{{{{{\rm{Cl}}}}}}}_{2({{{{{\rm{g}}}}}})}$$

This mechanism was based on laboratory observations of the production of 10–100 ppbv of Cl_2_ when gaseous O_3_ (0.8–14 ppmv) and deliquesced sea salt particles were illuminated with 254 nm ultra-violet light^27^. The experimental results were supported by molecular dynamics and kinetics calculations^28^. These studies revealed a maximum Cl_2_ production of 375 pptv s^−1^ (with 14 ppmv O_3_ and a photolysis rate constant for O_3_ to generate O(^1^D) (J(O_3_→O(^1^D)) of 7.92 × 10^−4^ s^−1^). This production rate was extrapolated to typical mid-Atlantic conditions, assuming that the Cl_2_ production was proportional to the level of ozone and solar radiation and Cl^−^ availability was sufficient. These conditions explained the observed Cl_2_ at a coastal site in Long Island, New York^27^. If we extrapolate their production rate, with the same assumptions, to our ambient conditions i.e., O_3_ (65 ppbv) and J(O_3_→O(^1^D)) (1.78 × 10^−5^ s^−1^), which is calculated from the TUV model under clear sky condition, the O_3_ photolysis would produce Cl_2_ at a rate of 0.039 pptv s^−1^, which is one order of magnitude smaller than the average daytime (08:00–18:00) production rate (P(Cl_2_)) of 0.46 pptv s^−1^ measured at our site. Here the P(Cl_2_) is assumed to be equal to the photolysis rate of Cl_2_, as the Cl_2_ is nearly in a photo-stationary state (considering its short lifetime of ~7 min at noon in our study).

Another suggested mechanism is the classic autocatalytic halogen activation, which begins with a Cl atom reacting with O_3_ to form chlorine monoxide (ClO) during daytime (R10–R11). ClO further reacts with HO_2_ or NO_2_ to form hypochlorous acid (HOCl) (R12) or chlorine nitrate (ClONO_2_) (R13), respectively. These two compounds can then undergo photolysis or react on acidic chloride-containing aerosol particles to form Cl_2_ (R14–R15) that partitions to the gas phase^9,29^.

R10 $${{{{{{\rm{Cl}}}}}}}_{2({{{{{\rm{g}}}}}})}+{{{{{\rm{h}}}}}}{{{{{\rm{\upsilon }}}}}}\to {{{{{{\rm{2Cl}}}}}}}_{({{{{{\rm{g}}}}}})}$$

R11 $${{{{{{\rm{Cl}}}}}}}_{({{{{{\rm{g}}}}}})}+{{{{{{\rm{O}}}}}}}_{3({{{{{\rm{g}}}}}})}\to {{{{{{\rm{ClO}}}}}}}_{({{{{{\rm{g}}}}}})}+{{{{{{\rm{O}}}}}}}_{2({{{{{\rm{g}}}}}})}$$

R12 $${{{{{{\rm{ClO}}}}}}}_{({{{{{\rm{g}}}}}})}+{{{{{{\rm{HO}}}}}}}_{2({{{{{\rm{g}}}}}})}\to {{{{{{\rm{HOCl}}}}}}}_{({{{{{\rm{g}}}}}})}+{{{{{{\rm{O}}}}}}}_{2({{{{{\rm{g}}}}}})}$$

R13 $${{{{{{\rm{ClO}}}}}}}_{({{{{{\rm{g}}}}}})}+{{{{{{\rm{NO}}}}}}}_{2({{{{{\rm{g}}}}}})}+{{{{{\rm{M}}}}}}\to {{{{{{\rm{ClONO}}}}}}}_{2({{{{{\rm{g}}}}}})}+{{{{{\rm{M}}}}}}$$

R14 $${{{{{{\rm{HOCl}}}}}}}_{({{{{{\rm{g}}}}}})}+{{{{{{\rm{H}}}}}}}^{+}+{{{{{{\rm{Cl}}}}}}}^{-}\mathop{\longrightarrow }\limits^{aerosol}{{{{{{\rm{Cl}}}}}}}_{2({{{{{\rm{g}}}}}})}+{{{{{{\rm{H}}}}}}}_{2}{{{{{{\rm{O}}}}}}}_{({{{{{\rm{aq}}}}}})}$$

R15 $${{{{{{\rm{ClONO}}}}}}}_{2({{{{{\rm{g}}}}}})}+{{{{{{\rm{H}}}}}}}^{+}+{{{{{{\rm{Cl}}}}}}}^{-}\mathop{\longrightarrow }\limits^{aerosol}{{{{{{\rm{Cl}}}}}}}_{2({{{{{\rm{g}}}}}})}+{{{{{{\rm{HNO}}}}}}}_{3({{{{{\rm{aq}}}}}})}$$

We used a photochemical box model^6^ (also see Methods section “Chemical box model”) to simulate HOCl and ClONO_2_ (Supplementary Fig. 3) based on known gaseous chlorine chemistry, by constraining it to the observed Cl_2_ and other chemical constituents concentrations (Supplementary Table 1 and Supplementary Fig. 4). The calculations were performed for the period 4—14 September 2018, for which a more complete VOC dataset is available. The simulated mixing ratios of HOCl were a factor of 3 lower than those of Cl_2_ (Supplementary Fig. 3), as Cl atoms predominantly react with volatile organic compounds (VOCs) (~83%) but less efficiently with ozone (~17%) to form ClO and then HOCl at our site (see below). The calculated Cl_2_ production rate (via R14) was two orders of magnitude lower than the observed rate, even if we adopt the highest model-predicted HOCl value (180 pptv) and previously reported the highest HOCl uptake coefficient of 0.0002 (Methods section “Estimation of Cl2 production from heterogeneous reactions of HOCl”), confirming the negligible role of HOCl in producing Cl_2_ (via R14) at our site. For ClONO_2_, the model calculated mixing ratios (Supplementary Fig. 3) were two orders of magnitude lower than the observed Cl_2_ values, suggesting its insignificant role in Cl_2_ production (via R15). To conclude, the previously two known mechanisms for producing daytime Cl_2_ cannot account for the high Cl_2_ production observed, and the mismatch is larger than an order of magnitude.

To gain more insight into the potential sources of daytime Cl_2_, we examined the relationship between P(Cl_2_) and various measured parameters (see Supplementary Fig. 5) that might be involved in the Cl_2_ photochemical production. We found a good correlation between P(Cl_2_) and the product of the solar actinic flux and the aerosol surface area density (*R*^2^ = 0.71) (Supplementary Fig. 5), and the correlation was further improved with consideration of O_3_ (*R*^2^ = 0.91) (Fig. 1C) or nitrate in aerosol (*R*^2^ = 0.90) (Fig. 1D). The high correlation between the product of O_3_ abundance and surface area density and P(Cl_2_) is not necessarily the result of a causal relationship between O_3_ and Cl_2_, but likely highlights their photochemical co-production. In other words, we suggest that this is a consequence of the chemistry rather than the cause of the Cl_2_ production.

Our observations suggest that photochemistry on the particle surfaces is the important driver of the high Cl_2_. The strong correlation between P(Cl_2_) and the product of nitrate and aerosol surface area density suggests that photolysis of nitrate-laden particles may be involved in the chloride activation to produce Cl_2_ at our site. (Note that the correlation was largely decreased (*R*^2^ = 0.39) if the surface area density was excluded). The Cl_2_ production via chloride activation also requires particulate chloride. Interestingly, the average chloride (Cl^−^) concentrations were comparable in the oceanic air with low Cl_2_ (0.56 μg m^−3^ in PM_2.5_ and 2.47 μg m^−3^ in PM_10_) and the continental air mass (0.50 μg m^−3^ in PM_2.5_, 2.38 μg m^−3^ in PM_10_). The average Cl/Na mass ratio in the oceanic air was 1.48 in PM_2.5_ and 1.63 in PM_10_ compared to 1.10 in PM_2.5_ and 1.33 in PM_10_ in continental air, indicating that Cl was more depleted in polluted air than in the clean air, in comparison to their average ratio of 1.8 in seawater^30^. These results suggest that Cl^-^ was not the limiting factor, and the Cl_2_ production was mainly controlled by nitrate availability and other factors.

### Laboratory investigations of Cl_2_ production

To explore the photochemistry leading to Cl_2_ production, a series of experiments were undertaken by illuminating nitrate and chloride-containing solutions and ambient aerosols in the presence of gaseous O_3_ with a high-pressure xenon lamp (Supplementary Fig. 6). The experimental setup and detailed information (designs, lamp, and chemicals) are given in Methods section “Lab Experiments” (and Supplementary Fig. 7). The average relative humidity (RH) during the field measurements was 81%, which was above the deliquescence point of sodium chloride (75%), and thus a very large fraction of sea-salt aerosols should have been wet during our field study. We, therefore, investigated Cl_2_ production over or in solutions.

No Cl_2_ was observed in the blank experiments, which were run with an empty chamber or with a quartz petri dish containing deionized water or chloride placed in the chamber, in the dark, or when illuminated by the xenon lamp. We also did not detect Cl_2_ when zero air containing various O_3_ mixing ratios (150, 250, and 500 ppbv) flowed over the illuminated solution of 1 M sodium chloride. The result shows that O_3_ photolysis alone does not produce any detectable amount of Cl_2_ in our experiment, as observed previously^27^. We note that the rate constant for O_3_ to generate O(^1^D) (1.31 × 10^−5^ s^−1^) in our experiment was two orders of magnitude lower than that in the previous study^27^, who used a more intense UV light source.

**Fig. 2:**
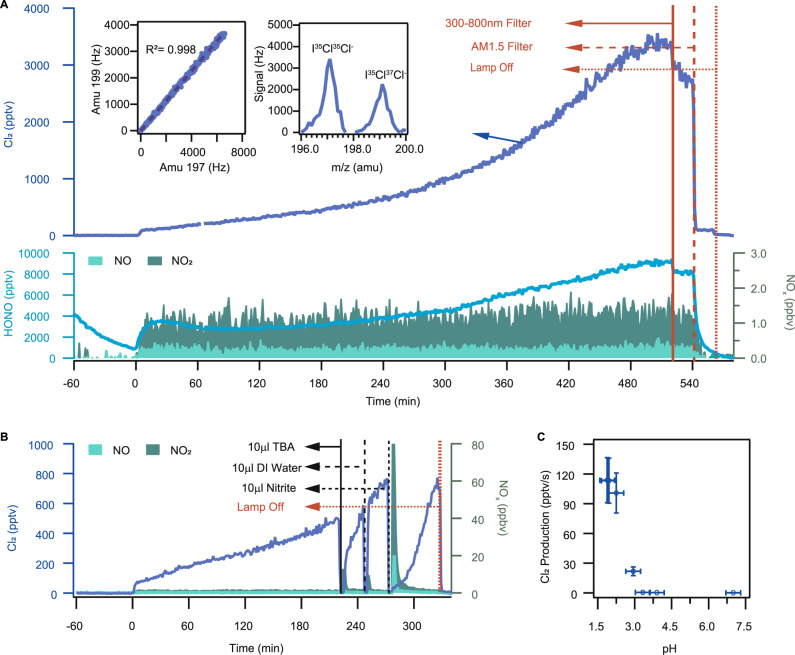
Experimental results on solutions in the dynamic chamber. **A** Time series of 1-min average mixing ratios of Cl_2_, HONO, and NO_x._ The liquid solution samples (pH = 1.9) were illuminated at *t* = 0. The solid red line shows the time at which 300–800 nm filter was used, the red dashed line indicates the time at which AM1.5 filter was used, and the red point line indicates the time at which the xenon lamp was turned off. The left inset: scatter plot of the raw CIMS signal of Cl_2_ at mass 199 atomic mass unit (amu) (I^35^Cl^37^Cl^−^; I^37^Cl^35^Cl^−^)) versus 197 amu (I^35^Cl^35^Cl^−^) with 1-min average from *t* = −60 to *t* = 580 min. The right inset: the scanned mass spectra from 196 amu to 200 amu at *t* = 387 min. The continuous increase of Cl_2_ may be due to the higher concentration of ions and acidity in the solution due to the evaporation of water from the solution. **B** Time series of 1-min average Cl_2_, NO, and NO_2._ The liquid solution samples (pH = 2.0) were illuminated at *t* = 0. The solid black line shows the time at which 10 μl OH scavenger, TBA, was added, the black dashed line indicates the time at which 10 μl DI water was added, the black point line indicates the time at which 10 μl nitrite was added, and the red point line indicates the time at which the xenon lamp was turned off. **C** The production rate of Cl_2_ as a function of initial solution pH (pH = 1.9; 2.0; 2.3; 2.9; 3.3; 3.9; 6.8) at the illumination time of 500 min. The error bars in the plot (**C**) represent the estimated uncertainty in Cl_2_ and pH measurement. Experimental conditions: 75−83% RH, 298 K in air and one 4 ml liquid solution containing 1 M NaCl + 1 M NaNO_3_.

Interestingly, we observed significant Cl_2_ production when acidic solutions (pH < 3.3) containing both chloride and nitrate were illuminated. Irradiation of the solution, with an initial pH of 1.9, led to a continuous increase of gaseous Cl_2,_ and up to 3.5 ppbv was observed after 500 min of illumination (Fig. 2A). Previous laboratory studies of halogen production under similar but not identical conditions (i.e., light source and reaction medium) indicated that reactive bromine gases (Br_2_ and BrCl) were produced over acid-doped nitrate-halide solution (liquid or frozen) under UV light (~310 nm)^31–33^, but Cl_2_ was not observed, unlike our experiment. Note that in our study, Br_2_ and BrCl were also produced together with Cl_2_. We also investigated the influence of ozone on Cl_2_ production. There was no relative increase in the Cl_2_ signals when zero air containing differing O_3_ mixing ratios (150, 250, and 500 ppbv) flowed over the illuminated chloride-nitrate solutions with a pH of 1.9 to 2.9, compared to the no O_3_ cases (Supplementary Fig. 8A for pH = 1.9). And the Cl_2_ level also did not increase when the added O_3_ increased from 150 ppbv to 500 ppbv with a pH of 3.3 to 6.8 (Supplementary Fig. 8B for pH = 3.9). When we placed an AM1.5 optical filter in front of the xenon lamp, which only allows the light with the wavelength > 360 nm to pass through, there was a sharp decrease in the Cl_2_ (and HONO) signals (shown at t = 540 min), whereas using a 300–800 nm optical filter (allowing the 300–800 nm light to pass through) only slightly decreased the Cl_2_ (and HONO) production (shown at *t* = 520 min). This result reveals large Cl_2_ production occurring at the wavelength of < 360 nm despite its concurrent significant photolytic loss.

Based on the above results, we propose that the hydroxyl radical (OH) from the nitrate photolysis and subsequent oxidation of chloride in solution was primarily responsible for the observed high rate of Cl_2_ production (R12–R14, R4, R5).

R16 $${{{{{{\rm{NO}}}}}}}_{3}^{-}+{{{{{\rm{h}}}}}}{{{{{\rm{\upsilon }}}}}}\,( < 350\,{{{{{\rm{nm}}}}}})\to {{{{{{\rm{O}}}}}}}^{-}+{{{{{{\rm{NO}}}}}}}_{2({{{{{\rm{aq}}}}}})}({{{{{\rm{R1}}}}}},\,{{{{{\rm{yield}}}}}} \sim 0.01)$$

R17 $$\to {{{{{{\rm{NO}}}}}}}_{2}^{-}+{{{{{\rm{O}}}}}}{({\!\,}^{3}{{{{{\rm{P}}}}}})}_{({{{{{\rm{aq}}}}}})}({{{{{\rm{R2}}}}}},\,{{{{{\rm{yield}}}}}} \sim 0.001)$$

R18 $${{{{{{\rm{O}}}}}}}^{-}+{{{{{{\rm{H}}}}}}}^{+}\rightleftharpoons {{{{{{\rm{OH}}}}}}}_{({{{{{\rm{aq}}}}}})}$$

It has been known that nitrate absorbs light in the actinic range of 290–350 nm and dissociates via two pathways (R16 and R17) with a quantum yield of 0.017 and 0.001, respectively^34–37^. O^−^ (produced from R16) reacts with water to form the hydroxyl radical (OH) (R18). This process can be accelerated by the acidity of the solution^34^. The produced OH can further oxidize Cl^−^ to produce Cl_2_ in the liquid phase (R4-R9), according to previously known aqueous chemistry^28,34,38^, and a portion of the Cl_2_ is released to the gas phase. Our observation of HONO and NO_2_ production (see Fig. 2A, B) supports that R16 and R17 were taking place in our experiment and is consistent with the previous studies showing the production of HONO and NO_2_ from illuminated nitrate solutions^39^. To confirm the role of aqueous OH radical in the Cl_2_ production, we added 10 µl 0.1 M Tert-Butyl Alcohol^40^ (TBA, a scavenger of OH radical scavenger with a rate constant of (3.8–7.6) × 10^8^ M^−1^ s^−1^) in the illuminated solution (Fig. 2B). There was a sharp decrease of the Cl_2_ signal lasting for 20 min before returning to the previous level. To make sure that this change was not caused by operation (i.e., opening the chamber), we added 10 μl Deionized (DI) water into the chamber, and the Cl_2_ signal bounced back in a few seconds. This result confirmed that the aqueous OH radical played a significant role in the Cl_2_ production in the chamber.

We found that the Cl_2_ production was strongly dependent on the acidity of the solution (Fig. 2C and Supplementary Fig. 9). The production rate sharply dropped to near zero when the pH increased from 2.9 to 3.3. It is expected that increasing pH would decrease the OH radical production via R18 and through the HOCl^−^ adduct (R3–R9), and hence decrease the rate of Cl_2_ production via R4–R9^28^. In addition, when the pH increases (H^+^ decreasing), nitrite ions (NO_2_^−^) would produce less HONO in the aqueous phase, which in turn produces less aqueous OH radical (via R19) and then Cl_2_. Interestingly, there seems a critical pH (~3.3) above which little Cl_2_ is produced. This can be explained by suppression of OH by NO_2_^−^ above this pH value of 3.3. The dissociation constant (pKa) of HONO at 298 K is 3.3^41,42^, i.e., above pH = 3.3, NO_2_^−^ is the predominant species in solution. We found that NO_2_^−^ can efficiently suppress OH concentration. When we added a very small amount of NO_2_^−^ (10 μl 0.01 M) in the illuminated solution, the concentration of Cl_2_ decreased significantly (Fig. 2B), revealing that NO_2_^−^ is an OH scavenger. Figure 2B shows that it took twice as long for the Cl_2_ signal to return to the previous level, compared to the case of TBA, suggesting that NO_2_^-^ is a more efficient OH scavenger than TBA. In summary, based on our experimental results, we hypothesize that the photolysis of nitrate has two different effects on Cl_2_ production. One is to promote Cl_2_ production by increasing OH (R4–R9), and the other is to inhibit Cl_2_ formation via nitrite. Increasing solution pH allows more NO_2_^−^ to stay in the solution and reduces Cl_2_ production.

R19 $${{{{{{{\rm{NO}}}}}}}_{2}}^{-}+{{{{{{\rm{H}}}}}}}^{+}\rightleftharpoons {{{{{\rm{HONO}}}}}}\,({{{{{\rm{aq}}}}}})\rightleftharpoons {{{{{\rm{HONO}}}}}}\,({{{{{\rm{g}}}}}})$$

We also investigated the effect of surface area on Cl_2_ production. In the laboratory experiments, more Cl_2_ was observed when 4 mL of the nitrate-NaCl solution was split into 4 × 1 mL samples (Supplementary Fig. 9). This may be explained by increased Cl_2_ production and diffusion into the gas phase from the increased surface area. Previous kinetic modelling^28^ and laboratory studies^43^ indicated preferential occupation of nitrate ions at the interface, which can facilitate fast surface reactions.

**Fig. 3:**
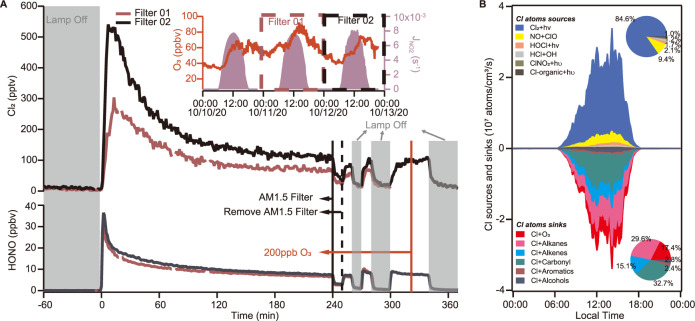
Experimental results on ambient aerosols and model results of Cl atoms budget. **A** Experimental results on ambient aerosols in the dynamic chamber. Time series of 1-min average Cl_2_ and HONO. Ambient samples (cropped size: 60 mm × 60 mm, Fig. S7C) were illuminated at *t* = 0. The grey area indicates the period, which the xenon lamp was turned off, the solid black line shows the time at which the AM1.5 filter was used, the black dashed line indicates the time at which the AM1.5 filter was removed, and the solid red line indicates the time at which 250 ppbv O_3_ was added. The right inset: ambient observations of O_3_ and J_NO2_ during the ambient aerosol collection in October 2020. Experimental conditions: 75−83% RH and 298 K in air. **B** The model-calculated average diurnal profiles of sources and sinks of the Cl atom for period 4−14 September 2018. Upper right inset: the daytime average contribution from different sources to Cl atom. Bottom right inset: the average daytime contribution from different sinks to Cl atom.

To further investigate the daytime Cl_2_ formation under ambient conditions, four aerosols samples collected (for 24-h duration each) at the same site on 11–13 October 2020 were irradiated in the dynamic chamber. As shown in Fig. 3A and Supplementary Table 2, Cl_2_ mixing ratios of up to 600 pptv were observed after illuminating two of the aerosol particle-loaded filters (filter 01 and 02) containing high concentrations of Cl^−^ and NO_3_^−^. Interestingly, the produced Cl_2_ were below the detection limit in the other two filters (filter 03 and 04) loaded with particles that contain low concentrations of Cl^−^ and NO_3_^−^. In filter 01, the high level of Cl_2_ was observed along with HONO, suggesting the potential role of particulate nitrate photolysis in their productions. Similar to the experiment performed on the Cl^−^ and NO_3_^−^ solution, the Cl_2_ levels decreased with the use of AM1.5 optical filter, indicating the wavelengths < 360 nm are very important for Cl_2_ (and HONO) production, and no increase in Cl_2_ was observed when we flowed the zero air containing 250 ppbv O_3_ (Fig. 3A).

We next attempt to extrapolate the laboratory results to account for the observed atmospheric daytime Cl_2_. The field observations and laboratory experiments suggest that the Cl_2_ production likely occurs on the aerosol surface. The pH for most aerosol samples (>90%) in the 2018 field campaign was in the range of 1–3, with an average value of 1.5 (Supplementary Fig. 10), which was estimated from the E-AIM model (see Methods section “HYSPLIT and E-AIM models”). The laboratory-determined Cl_2_ production rates on liquid solutions at pH of 1.9 (similar to the average value of the ambient aerosol), and 1 mol L^−1^ nitrate was 114 pptv s^−1^ (shown at *t* = 520 min in Fig. 2). The surface area density of the solution in the chamber air is 5.13 × 10^5^ μm^2^ cm^−3^ (see Methods section “Lab Experiments”), which gives a Cl_2_ production rate of 214 mol m^−2^. For the continental air mass and during the period of 10:00–15:00, the average surface area density of ambient aerosols was 653 μm^2^ cm^−3^ after taking into account aerosol hygroscopicity when assuming the particles to be spherical, and the nitrate concentration in the aerosol liquid phase estimated by the E-AIM model was 3.9 mol L^−1^. This gives an estimated Cl_2_ production of 0.57 pptv s^−1^, which could explain 68% of the observed average Cl_2_ production rate (~0.84 pptv s^−1^) in the ambient air. In addition to the airborne aerosol, the Cl_2_ production may occur on the aerosols deposited on the ground, which could provide additional production that would help to reconcile the lab and field observed Cl_2_ productions. We note that the above extrapolation is subject to uncertainty, including that from applying the Cl_2_ production over the tested solution to the ambient aerosol, that from estimating aerosol pH with current aerosol thermodynamic models, and not accounting for competing reactions for OH and Cl, such as by organics. It is also possible that other unidentified source(s) may contribute to part of the observed daytime Cl_2_.

The above results indicate the importance of the coexistence of the three key factors in Cl_2_ production, namely, nitrate, chloride, and acidity in the aerosol particles. Our proposed Cl_2_ production mechanism could qualitatively explain the lack of daytime Cl_2_ in previous studies that reported lower aerosol acidity or lower aerosol chloride content. During shipborne measurements off the coast of Los Angeles, elevated Cl_2_ concentrations were observed mostly at night and in isolated industrial plumes^15^. Their E-AIM model calculated sub-micrometer aerosol pH was > 4. According to the pH-dependency of Cl_2_ production in our experiments, the Cl_2_ production is expected to be very slow under such conditions, which could explain the absence of daytime Cl_2_ in their study. Very low levels of ClNO_2_ (and lack of Cl_2_) were reported in the oil-exploration impacted Uintah basin^44^, and the author attributed the low ClNO_2_ to lacking aerosol-phase chloride at this remote inland site, which would also explain the absence of Cl_2_ in that study.

Our result has shown that pH below 3.3 is a necessary condition for nitrate-induced Cl_2_ production. There could be a lower limit of pH for this production pathway. If the pH were too low, Cl^−^ would be converted to HCl and NO_2_^−^ to HONO, the former would reduce Cl_2_ production while the latter would promote Cl_2_ formation. Accordingly, there could be a window of suitable pH conditions for the described way of Cl_2_ formation to efficiently take place. The estimated average pH for PM_2.5_ at our study site was 1.6 (range: 0.5–3.0), and a very large production of Cl_2_ was observed under such acidic conditions. A recent review of global aerosol acidity^45^ indicates that about 90% of fine-mode aerosol has a pH larger than 0.5, with 80% above 1.6. We suggest that the lower pH limit at which Cl_2_ production would decrease may not be reached for typical tropospheric aerosols. That is, aerosol pH smaller than 3.0 should promote Cl_2_ production under most tropospheric conditions.

### Impact on atmospheric chemistry and implications

We assess the effects of the observed Cl_2_ on VOC oxidation using a photochemical box model with up-to-date VOC-Cl chemistry^6^ (also see Methods section “Chemical box model”) by constraining the model with the measured Cl_2_ abundance and other relevant observations for 4–14 September 2018. The average values and the diurnal profiles of the input data indicate moderately polluted conditions for this period, with an average peak mixing ratio of O_3_ of ~80 ppbv and Cl_2_ of 300 pptv and NO_x_ and VOCs levels characteristic of the polluted rural environment (Supplementary Table 1, and Supplementary Fig. 4). The model predicted that Cl atoms reached a maximum concentration of ~2.5 × 10^5^ cm^−3^ at noon (Supplementary Fig. 11A), with Cl_2_ photolysis being the dominant source (~85%) (Fig. 3B). The peak Cl production rate at our site (~4 × 10^7^ cm^−3^s^−1^, Fig. 3B) is more than five to six times of that from the photolysis of BrCl and Cl_2_ in winter^6^ or from the photolysis of Cl_2_ (predominantly) and ClNO_2_^19^ in summer in a rural area of northern China, and is two orders of magnitude larger than that from the photolysis of Cl_2_ and ClNO_2_ in late autumn and early winter at a ground site near the City of Manchester, UK^20^.

The Cl atoms accounted for 59% of daily integrated oxidation of non-methane alkanes, 16% of aromatics, 13% of aldehydes, and 9% of dialkenes (Supplementary Fig. 12). The reactions of Cl atoms with VOCs produce RO_2_ radicals, which are recycled to form HO_2_ and OH radicals, thereby collectively increasing the average mixing ratios of OH, HO_2_, RO_2_ radicals by ~4%, ~17%, and ~27%, respectively (Supplementary Fig. 11). The enhanced HO_2_ and RO_2_ by Cl-VOC reaction increased the in-situ net total ozone (O_x_, O_3_ + NO_2_) production rates by 17% (or 1.6 ppbv h^−1^) and its daily integrated production by 16% (or 38 ppbv day^−1^) (Supplementary Fig. 11), despite destroying ozone by Cl atoms at the same time (Fig. 3B). With a high-resolution time of flight mass spectrometer, we also observed elevated concentrations of organochlorides (e.g., 1-chloro-3-methyl-3-butanone, CMBO) with a similar diurnal profile to Cl_2_, a possible indication of significant oxidation of VOCs by chlorine atoms (Supplementary Fig. 13). These results demonstrate the substantial impact of Cl_2_ on daytime oxidation chemistry at our moderately polluted site.

In summary, a limited number of prior studies have indicated the presence and important role of daytime Cl_2_ in the photochemistry of the lower troposphere in polluted regions. However, the exact source or production mechanism remained uncertain, which has hindered the reproduction of the daytime Cl_2_ in current state-of-the-art global and regional chemistry transport models. In the present study, we observed very high Cl_2_ concentrations at a polluted coastal site, implying that the Cl_2_ could exist in more places and at higher concentrations than previously thought, in view of the limited Cl_2_ observations to date. Combining laboratory and field measurements, we show that Cl_2_ can be produced from photolysis of aerosol containing nitrate, chloride, and high acidity, and demonstrate that this mechanism can explain a great fraction of the observed daytime Cl_2_ at our measurement site. Our result indicates the critical role of aerosol acidity (pH < 3.3) in promoting Cl_2_ production. The same mechanism may occur in other parts of the world impacted by anthropogenic pollution. Despite a significant reduction in the emissions of acid precursors like sulfur dioxide (SO_2_) and nitrogen oxides (NO_x_), highly acidic aerosols are still present in some areas/seasons in Asia, North America, and Europe^45^, and nitrate aerosols are also abundant in world’s urban and industrial regions^46,47^. Previous studies have also indicated the ubiquity of aerosol chloride in continental as well as maritime environments^3,30,48,49^. We, therefore, anticipate that the Cl_2_ production operates in some places or times where/when sufficiently high levels of acidity, nitrate, and chloride co-exist. Our recent study in northern China also shows that nitrate photolysis could activate chloride and bromide in coal-burning aerosol, which exerted a large impact on winter oxidation chemistry^6^. We note that elevated nitrate and the other acidic aerosol (sulfate) have been observed during the Arctic haze events^50,51^. It would be of great interest to investigate whether the nitrate photolysis mechanism would contribute to the liberation of inert chlorine in the polar troposphere.

Our findings have indicated a previously unrecognized role of the reactive nitrogen cycle in both halogen and HO_x_ chemistry and, at the same time, an interesting coupling between condensed phase oxidation resulting in the formation of its gaseous counterpart, which is expected to have important implications on atmospheric chemistry and production of secondary air pollutants. These findings suggest a direction for developing the Cl_2_ scheme to predict its impact on oxidation chemistry for air quality models that currently do not include such chemistry. Moreover, our results suggest an additional benefit of widely adopted antipollution measures to control SO_2_ and NO_x_. That is, reducing SO_2_ and NO_x_ emissions not only alleviates their adverse impacts on health and welfare directly and through the formation of acid deposition and particles, but also decreases particle acidity and nitrate, both of which would slow the Cl_2_ production and its promoting consequences to secondary pollution production, for example, surface ozone. We call for more investigations of the roles of halogen chemistry in the polluted troposphere and suggest some research that would place Cl_2_ (and other halogens) production and atmospheric impact on a firmer footing. They include more atmospheric measurements of Cl_2_ together with aerosol acidity (or its proxies), nitrate, and other parameters in diverse geographical areas, detailed laboratory measurements of the photolysis of nitrate ion in aerosol as a function of acidity, complete characterization of the aerosol particles to identify the origin of chloride in them, robust ways to determine rates of photolysis in and on aerosol particles, and parameterization of Cl_2_ production to assess the broader impact of reactive chlorine chemistry in regional and global models.

## Methods

### Field measurements

Cl_2_, ClNO_2_, HONO, NO, NO_2_, O_3_, volatile organic compounds (VOCs), oxygenated volatile organic compounds (OVOCs), aerosol compositions (including/e.g., Na^+^, NH_4_^+^, SO_4_^2−^, NO_3_^−^, Cl^−^), solar radiations, and other meteorological parameters were measured from 31 August to 09 October of 2018 at the Hong Kong Environmental Protection Department’s Cape D’Aguilar Super Site, which is situated at the southeast corner of Hong Kong Island (Supplementary Fig. 1). We introduce in detail Cl_2_ and other species measured by a chemical ionization mass spectrometer (CIMS). Information on other measurements is summarized in Supplementary Table 3.

Reactive chlorine species (including Cl_2_, ClNO_2_, and HOCl) and HONO were measured by a quadrupole CIMS (Q-CIMS). A detailed description of the CIMS and ion chemistry has been described in our previous studies^6,17^. Briefly, Iodide (I^−^) was used as a reagent ion. Cl_2_ was monitored at 197 amu (I^35^Cl^35^Cl^−^) and 199 amu (I^35^Cl^37^Cl^−^), ClNO_2_ at 208 amu (I^35^ClNO_2_^−^) and 210 amu (I^37^ClNO_2_^−^), HOCl at 179 amu (IHO^35^Cl^−^) and 181 amu (IHO^37^Cl^−^), and HONO at 174 amu. In this study, we used the data of Cl_2_, ClNO_2_, and HONO from CIMS measurement. The HOCl signals suffered from spectral interference, as indicated by the weak correlation between the two isotopic masses, and thus were not used in further analysis.

The instruments were housed in a one-story building. The inlet is a 3.5-m long PFA-Teflon tubing (1/2 in. outer diameter) with 1.5 m of it situated above the roof. We adopted the previous inlet design as described in our previous study^6^. To further reduce the residence time (and thereby potential artifacts) in the inlet tubing, we used a blower with a flow rate of 500 SLPM flow to draw the sample. As a result, the residence time of the sample air in the inlet tubing was below 0.1 s. To reduce the particle deposited on the inlet tubing, the tubing was flushed with DI water and then dried by drawing ambient air for 20 min every three days.

The following on-site and post-measurement measures were undertaken to ensure accurate measurements of Cl_2._ They included (1) Instrument background determination. During the study, the CIMS background signals were determined about every two days by scrubbing ambient air with alkaline glass wool and charcoal^6^. Many inorganic halogens are efficiently removed by this process. In the present study, the background for Cl_2_ was small and relatively stable at around 5 pptv during the field campaign (Supplementary Fig. 14B). The 2-σ detection limit was 9 pptv for Cl_2_ (at 197 amu). (2) Regular calibration with a Cl_2_ standard. The calibration of Cl_2_ was conducted on-site every 2–4 days with a Cl_2_ permeation tube. The detailed calibration methods have been described in our previous study^6^. The permeation rate of the Cl_2_ standard was determined before and after the campaign and was stable at around 210 ng min^−1^, with a variation of less than 5% during the field campaign. The sensitivity of Cl_2_ was stable at 2.0 Hz pptv^−1^ with a standard deviation of 0.2 Hz pptv^−1^, as shown in Fig. S14D. The Cl_2_ sensitivity was invariant at RH of 20–80% (Supplementary Fig. 14E). The measurement uncertainty for Cl_2_, calculated from the variation of the sensitivity during the campaign and the uncertainty of permeation tube source, was about 11%. (3) Examination of the isotopic ratio of the detected compounds. During the field study, we confirmed that the detected signal for Cl_2_ had no significant spectral interference. The two isotopic masses at 197 amu and 199 amu were well resolved, as shown in Supplementary Fig. 14A, and showed excellent correlation (*R*^2^ = 0.93) with a slope of 0.63 (Supplementary Fig. 14C), which is similar to the theoretical value of 0.65. (4) Investigation of conversion on inlet surfaces. We conducted a series of tests to examine potential inlet interferences in the field and laboratory, which included: (i) potential inlet artifact arising from heterogeneous reactions of ambient O_3_ and N_2_O_5_ were tested by adding O_3_ and N_2_O_5_ into the ambient air in the intake to the measurement system. Cl_2_ often coincided with high O_3_ concentrations in the field, and previous lab^52^ and field studies^17^ indicate potential Cl_2_ formation involving N_2_O_5_. During the field sampling period, when we turned off the bypass blower and injected concentrated O_3_ and N_2_O_5_ into the ambient air sample (resulting in 250 ppb of O_3_ and 5 ppbv of N_2_O_5_ after mixing with the ambient air), we did not observe any increased Cl_2_ signals. This result indicates that the O_3_ and N_2_O_5_ did not produce detectable artifacts in our Cl_2_ measurement. (ii), potential inlet artifact tests of HOCl reactions in the laboratory. A previous study reported that 15% of the HOCl was lost in their NaCl-coated inlet, with 2% converting to Cl_2_^11^. Our post-campaign tests confirmed that the observed Cl_2_ did not suffer from significant interference from HOCl in the sampling inlet. Briefly, we tested two types of Teflon tubing: one used in the campaign and a new tubing with the same length. HOCl was synthesized using a phosphate-buffered solution (pH = 6.8) of NaOCl (11–14% chlorine, Alfa Aesar) and AgNO_3_, analogous to the previous method^11,14^. A 20 sccm dry N_2_ was flowed through the solution and then diluted into 6 SLPM humidified zero air. The concentration of HOCl was calculated from the Cl_2_ formation by passing the HOCl standard through a NaCl-coated tubing. For the HOCl inlet conversion test, the synthesized HOCl mixed with 6 SLPM humidified zero air was first introduced to the CIMS without passing through the tubing. Then, the HOCl/air mixture passed through the tubing before entering the CIMS. The decrease in the HOCl signal and the increase in the Cl_2_ signal induced by the tubing were monitored to determine the conversion of HOCl to Cl_2_ in the tubing. Under the RH condition similar to the field campaign, we found that 31% and 7% of the HOCl were lost, and 18% and 2% were converted to Cl_2_ in the used tubing and the new tubing, respectively. As the flow rate in the laboratory (6 SLPM) was much lower than the ambient sample flow rate (500 SLPM), we conclude that the conversion rate of HOCl to Cl_2_ during the field measurement should be much lower than 18%.

### HYSPLIT and E-AIM models

Three‐day (72 h) backward trajectories were calculated for each hour using the Hybrid Single‐Particle Lagrangian Integrated Trajectory (HYSPLIT) model (https://www.ready.noaa.gov/HYSPLIT.php). The HYSPLIT was driven by 3‐hourly archive data from NCEP’s GDAS with a spatial resolution of 1 degree by 1 degree. The endpoint of the trajectories was 300 m above ground level at Hok Tsui, which is in the middle of the marine boundary layer. Air masses were then classified based on the source regions (ocean or continent).

The H^+^ concentrations ([H^+^], in mol L^−1^) in the aqueous phase of aerosols were calculated using the E-AIM model (E-AIM III) online (http://www.aim.env.uea.ac.uk/aim/model3/model3a.php)^17,53^. The inputs to the model are hourly measurements of ambient RH and molar concentrations (unit: mol m^−3^) of Cl^−^, NO_3_^−^, SO_4_^2−^, Na^+^, and NH_4_^+^ in PM_2.5,_ which were measured by an ion chromatography (MARGA, Supplementary Table 3) and gas-phase ammonia. Aerosol pH was estimated as the negative logarithm of [H^+^] without further consideration of the activity coefficient of ions in the aqueous phase.

### Chemical box model

A zero-dimensional gas-phase chemical box model was used to calculate the budget for Cl atoms and to evaluate the observed Cl_2_ on atmospheric oxidation. The detailed information on the mechanisms and their related kinetics data of gas-phase reactions adopted in the model is given in the previous study^6^. The measured values of Cl_2_, ClNO_2_, N_2_O_5_, HONO, O_3_, NO, NO_2_, SO_2_, CO, and temperature were averaged or interpolated every minute and constrained into the model. The measured VOCs and OVOCs (except for CH_4_ and HCHO) were interpolated every minute and constrained into the model. The mixing ratio of CH_4_ was kept at a constant value of 2000 ppbv^54^. As the HCHO measurement data was not available in the 2018 field campaign, we used the HCHO measurement data obtained during September 2020 by off-line DNPH-Cartridge-HPLC (24h-average, 3.3 ppb) and adjusted for its diurnal variation according to a typical HCHO profile in a non-urban environment^55^. The input data for HCHO is shown in Supplementary Fig. 4.

The photolysis frequencies for Cl_2_, HONO, O_3_, and other species were calculated from the TUV model (http://cprm.acom.ucar.edu/Models/TUV/Interactive_TUV/) under clear sky condition and then scaled to J_NO2,_ which was derived from the measured solar radiation and relationship with J_NO2_ for Guangzhou (~100 km north of the present site)^56^. The dry deposition process in the model was represented by a first-order loss reaction, using the same parameter described in the previous study^57^. The boundary layer height was set at 200 m at nighttime and 1500 m for daytime in the model. The wet deposition was ignored as no rainfall event occurred during the observation period. The model was run from 00:00 of 4 September to 00:00 of 14 September, and the simulation for the first 24 h was repeated three times to stabilize the intermediate species. A summary of the input parameters in the model is shown in Table. S1, and the diurnal patterns of select input data are shown in Supplementary Fig. 4.

### Estimation of Cl_2_ production from heterogeneous reactions of HOCl

Cl_2_ can be produced from heterogeneous reactions of gaseous HOCl on a chloride-containing solution, with an uptake coefficient of HOCl up to 0.0002^22,58^. We used the following Eq. (1) to estimate the Cl_2_ production rate from the HOCl heterogeneous reaction.

1 $${{{{{\rm{The}}}}}}\,{{{{{{\rm{Cl}}}}}}}_{2}\,{{{{{\rm{production}}}}}}\,{{{{{\rm{rate}}}}}}=\frac{d[{{{{{\rm{HOCl}}}}}}]}{{dt}}=\frac{1}{4}{c}_{{{{{{\rm{HOCl}}}}}}}\gamma {S}_{a}[{{{{{\rm{HOCl}}}}}}]$$

Where *c* is the mean molecular speed of HOCl, *γ* is the heterogeneous uptake coefficient of HOCl, [HOCl] is the model simulated concentration, and *S*_*a*_ is the aerosol surface area density.

### Lab Experiments

#### The laboratory design

A dynamic reaction chamber was used to measure the productions of Cl_2_ by illuminating nitrate-NaCl solution and aerosol collected on filters. The overall experimental setup is shown in Supplementary Fig. 7 and is described here. The chamber is made of TFE Teflon (1.875 L, 25 cm-length × 15 cm-width × 4 cm-height) with a TFE Teflon-film window on the top. A quartz petri dish (inner diameter: 35 mm, internal height: 7 mm) held 4-ml liquid solution or filter samples. The surface area density of the chamber was determined as the physical surface area of the solution in the petri dish divided by the chamber’s volume and was 5.1 × 10^5^ μm^2^ cm^−3^. Zero air (2.9 SLPM) with adjustable humidity (75–83%) flowed through the chamber. The experiments were conducted at room temperature (296 K). A flow of O_3_ was diluted by zero air and then added into the chamber with the resulting O_3_ mixing ratio in the chamber ranged from 0 to 500 ppb. The residence time of the zero air/O_3_ was 0.625 min in the chamber. The outflow of zero air carrying the reaction products was monitored in real-time by the same iodide-CIMS instrument used in the field for Cl_2_ (amu 197, 199) and HONO (amu 174) detection and by a chemiluminescent/photolytic converter for NO and NO_2_.

To mimic the spectrum of the solar radiation, a high-pressure xenon lamp was used as the light source, and its spectral irradiance is shown in Supplementary Fig. 6. It covers from 320 nm to 1100 nm and peaks at 450 nm. Compared to the solar irradiance at a solar zenith angle of 48.2° (i.e., an air mass factor of 1.5 and standard ozone column abundance), the xenon lamp has a smaller flux in the range of 300 nm–326 nm but a larger flux in the range of 326 nm–420 nm. The photolysis rate constant for O_3_ to generate O^1^D (1.31 × 10^−5^ s^−1^) was similar to the daytime averaged rate constant of 1.78 × 10^−5^ s^−1^ (calculated from the TUV model under clear sky conditions) in the ambient air at our site (see Methods section “The determination of the photolysis rate and production rate of Cl_2_”), and the photolysis rate constant of Cl_2_ (${J}_{{{Cl}}_{2}}$) in our chamber (5.80 × 10^−3^ s^−1^, see below) was about four times larger than daytime averaged rate constant of 1.20 × 10^−3^ s^−1^ (calculated from the TUV model under clear sky conditions) in ambient air at our site. To investigate the role of photon energies, two optical filters were used (one is a 300–800 nm filter, which let the light with a wavelength of 300 - 800 nm to pass through, and the other, AM1.5 filter, which allows the light with the wavelength > 360 nm to go through).

To investigate the potential production of Cl_2_ in chloride and nitrate-containing solution, sodium chloride (NaCl, ACS, > 99.8%) and sodium nitrate (NaNO_3_, Sigma-Aldrich, >99.0%) were used as the source of particulate chloride and nitrate, respectively. Both NaCl and NaNO_3_ were prepared as 1 M L^−1^, which was similar to the average concentration of aqueous phase chloride and nitrate in ambient aerosols in the field study, which was estimated from the E-AIM (see above). The pH was adjusted by adding sulfuric acid (H_2_SO_4_, Sigma-Aldrich, 95–97%) and measured with a digital pH meter (HANNA instrument, HI253). In the experiments on the ambient filters, the aerosols of PM_2.5_ collected on quartz fiber filters with a high-volume sampler (Flow: about 890 L min^−1^, sampling period: 23.5 h, size: A4 page) were placed in the chamber.

#### The CIMS measurements in the laboratory

As was done in the field study, we conducted instrumental background checks, isotope analysis, and daily Cl_2_ calibration in the laboratory experiments. The background for Cl_2_ and HONO was stable. The sensitivity of Cl_2_ was stable at around 1.9 Hz pptv^−1^ with a standard deviation of 0.1 Hz pptv^−1^. HONO was calibrated at the end of the lab experiment. The sensitivities for HONO during the laboratory studies were determined according to its sensitivity ratio relative to that for Cl_2_. The sensitivity HONO was 3.0 Hz pptv^−1^. The measurement uncertainty for Cl_2_, calculated from the propagation of relative standard deviation for 1-min average data and the variation of the sensitivity within 1 day based on the calibration from permeation tube source, was about 5%. And measurement uncertainty for HONO, calculated from the propagation of both relative standard deviation for 1-min average data and the variation of the sensitivity, was about 15%.

#### The determination of the photolysis rate and production rate of Cl_2_

The photolysis rate of Cl_2_ (${J}_{{{Cl}}_{2}}$) in the chamber was calculated using the following Eq. (2)

2 $${J}=\int q(\lambda )\sigma (\lambda )I(\lambda )d\lambda$$

Where *q(λ)* is the quantum yield at wavelength *λ* (nm), *σ(λ)* is the cross-section of Cl_2_ at wavelength *λ*, which is adopted from the recommended value by IUPAC (http://iupac.pole-ether.fr/index.html). *I(λ)* is the flux of xenon lamp at wavelength *λ* and was calculated by converting the irradiation energy spectra of the lamp (Supplementary Fig. 6) to photon flux based on Planck’s equation. The same method was used to calculate the photolysis rate constant for O_3_ to generate O^1^D. The *q(λ)* and *σ(λ)* was adopted from the recommended value from IUPAC under 298 K (http://iupac.pole-ether.fr/index.html).

All laboratory experiments were carried out under the same light intensity with the same distance from the chamber (20 cm). As shown in Supplementary Fig. 7, almost the entire bottom area is illuminated by light. Under this configuration, the ${J}_{{{Cl}}_{2}}$ was estimated to be 5.80 × 10^−3^ s^−1^ without the optical filter, which was around four times larger than the daytime averaged photolysis rate constant of 1.20 × 10^−3^ s^−1^ in the ambient air. In calculation of ${J}_{{{Cl}}_{2}}$ in the chamber, we did not consider light reflection at the Teflon window and in the chamber inner surface as well as light loss during transmission. The calculated ${J}_{{{Cl}}_{2}}$was verified by another method, as shown in the end of this section.

The production rate of Cl_2_ (${P}_{{{Cl}}_{2}}$) in the dynamic chamber was determined based on the mass balance. The ${P}_{{{Cl}}_{2}}$ is equal to the sum of the photolysis loss rate of Cl_2_ and the advected loss of Cl_2_ in the dynamic chamber using the following Eq. (3):

3 $${P}_{{{Cl}}_{2}}={{{{{\rm{photolysis}}}}}}\; {{{{{\rm{rate}}}}}}\; {{{{{\rm{of}}}}}}\;{{{{{{\rm{Cl}}}}}}}_{2}+{{{{{\rm{advected}}}}}}\;{{{{{\rm{loss}}}}}}\;{{{{{\rm{of}}}}}}\;{{{{{{\rm{Cl}}}}}}}_{2}$$

Thus, (Cl_2_) (pptv s^−1^) = [Cl_2_] × ${J}_{{{Cl}}_{2}}+[{{{{{{\rm{Cl}}}}}}}_{2}]{{\times }}{{Q}}/{{V}}=[{{{{{{\rm{Cl}}}}}}} _{2}]{{\times }}(5.8{{\times }}{10^{-3}}{{{{{\rm{s}}}}}}^{-1}+2.7{{\times }}{10^{-2}}{{{{{\rm{s}}}}}}^{-1})$ under the experimental condition.

Where [Cl_2_] is the measured Cl_2_ mixing ratio (pptv), ${J}_{{{Cl}}_{2}}$ is the calculated photolysis rate (s^−1^), *Q* is the flow rate of the zero air thought the chamber (3 SLPM), and *V* is the volume of the chamber (1.875 L). Equation (3) assumes negligible Cl_2_ production from recombination of Cl atoms produced from Cl_2_ photolysis in the chamber, and this assumption is verified by the following experiments: we compared the Cl_2_ signals by the CIMS when 100 sccm a Cl_2_ standard was diluted by 2.9 SLPM zero air and further mixed with 100 sccm zero air or 100 sccm ozone-containing zero air (yielding 500 ppbv ozone in the chamber air). These experiments were conducted without the aerosol or liquid film in chamber. There was no detectable change in the Cl_2_ signals in the two tests (i.e., with or without ozone). This result confirms little Cl_2_ production from Cl back reaction, as the Cl_2_ signal with ozone added would have scavenged of Cl atom. Under the condition 3 SLPM flow (the condition of our experiments), advection was the predominant loss (accounting for 82%) of Cl_2_ produced in the chamber.

To verify the calculated ${J}_{{{Cl}}_{2}}$, we compared the Cl_2_ signals by the CIMS when the 100 sccm Cl_2_ standard diluted by 2.9 SLPM zero air (yielding 3.25 ppbv Cl_2_ in the chamber air) and then flowed through the chamber (without the condensed phase sample) with the lamp turned off and then on. The experimental results showed that there was a 16.5% drop in the Cl_2_ signals with the lamp on. Using the above equation for ${P}_{{{Cl}}_{2}}$ Eq. (3), the ${J}_{{{Cl}}_{2}}$ was determined at 5.3 × 10^−3^ s^−1^, which is very close to the calculated value (5.80 × 10^−3^ s^−1^) based on the lamp irradiance spectrum. Further, the calculated extent of Cl atom recombination is roughly consistent with this assertion of small loss of Cl atoms due to recombination. Based on these experiments and calculation, we suggest that the calculated ${J}_{{{Cl}}_{2}}$ is reliable.

## Supplementary information

Supplementary Information

Peer Review File

## Data Availability

The data that support the findings of this study are available in figshare with identifier (10.6084/m9.figshare.17099252).

## Code availability

The HYSPLIT model can be acquired from the NOAA Air Resources Laboratory website (https://www.ready.noaa.gov/HYSPLIT_linux.php). The source code of the chemical box model is available from 10.6084/m9.figshare.17099252.

## References

[1] Molina MJ, Rowland FS (1974). Stratospheric sink for chlorofluoromethanes: chlorine atomc-atalysed destruction of ozone. Nature.

[2] Molina LT, Molina MJ (1987). Production of chlorine oxide (Cl_2_O_2_) from the self-reaction of the chlorine oxide (ClO) radical. J. Phys. Chem..

[3] Thornton JA (2010). A large atomic chlorine source inferred from mid-continental reactive nitrogen chemistry. Nature.

[4] Wang T (2016). Observations of nitryl chloride and modeling its source and effect on ozone in the planetary boundary layer of southern China. J. Geophys. Res.: Atmospheres.

[5] Wang DS, Ruiz LH (2017). Secondary organic aerosol from chlorine-initiated oxidation of isoprene. Atmos. Chem. Phys..

[6] Peng, X. et al. An unexpected large continental source of reactive bromine and chlorine with significant impact on wintertime air quality. *Natl. Sci. Rev*. **8**, nwaa304 (2020). 10.1093/nsr/nwaa304 PMC8310770 34691692

[7] Li, Q. et al. Potential effect of halogens on atmospheric oxidation and air quality in China. *J. Geophys. Res. Atmos.* **125**, e2019JD032058 (2020). 10.1029/2019JD032058 PMC7286431 32523860

[8] Heimann M (2010). How stable is the methane cycle?. Science.

[9] Simpson WR, Brown SS, Saiz-Lopez A, Thornton JA, Glasow RV (2015). Tropospheric halogen chemistry: sources, cycling, and impacts. Chem. Rev..

[10] Solomon S, Garcia RR, Rowland FS, Wuebbles DJ (1986). On the depletion of Antarctic ozone. Nature.

[11] Liao J (2014). High levels of molecular chlorine in the Arctic atmosphere. Nat. Geosci..

[12] McNamara SM (2019). Springtime nitrogen oxide-influenced chlorine chemistry in the coastal arctic. Environ. Sci. Technol..

[13] Spicer CW (1998). Unexpectedly high concentrations of molecular chlorine in coastal air. Nature.

[14] Lawler MJ (2011). HOCl and Cl_2_ observations in marine air. Atmos. Chem. Phys..

[15] Riedel TP (2012). Nitryl chloride and molecular chlorine in the coastal marine boundary layer. Environ. Sci. Technol..

[16] Riedel TP (2013). Chlorine activation within urban or power plant plumes: Vertically resolved ClNO_2_ and Cl_2_ measurements from a tall tower in a polluted continental setting. J. Geophys. Res.: Atmospheres.

[17] Xia M (2020). Significant production of ClNO_2_ and possible source of Cl_2_ from N_2_O_5_ uptake at a suburban site in eastern China. Atmos. Chem. Phys..

[18] Impey GA, Shepson PB, Hastie DR, Barrie LA, Anlauf KG (1997). Measurements of photolyzable chlorine and bromine during the Polar Sunrise Experiment 1995. J. Geophys. Res.: Atmospheres.

[19] Liu X (2017). High levels of daytime molecular chlorine and nitryl chloride at a rural site on the North China Plain. Environ. Sci. Technol..

[20] Priestley M (2018). Observations of organic and inorganic chlorinated compounds and their contribution to chlorine radical concentrations in an urban environment in northern Europe during the wintertime. Atmos. Chem. Phys..

[21] Le Breton M (2018). Chlorine oxidation of VOCs at a semi-rural site in Beijing: significant chlorine liberation from ClNO_2_ and subsequent gas- and particle-phase Cl–VOC production. Atmos. Chem. Phys..

[22] Wang X (2019). The role of chlorine in global tropospheric chemistry. Atmos. Chem. Phys..

[23] Wang T, Lam K, Lee AS, Pang S, Tsui W (1998). Meteorological and chemical characteristics of the photochemical ozone episodes observed at Cape D’Aguilar in Hong Kong. J. Appl. Meteorol..

[24] Wang T, Dai J, Lam KS, Nan Poon C, Brasseur GP (2019). Twenty‐five years of lower tropospheric ozone observations in tropical East Asia: The influence of emissions and weather patterns. Geophys. Res. Lett..

[25] Dai J (2020). The impact of sea-salt chloride on ozone through heterogeneous reaction with N_2_O_5_ in a coastal region of south China. Atmos. Environ..

[26] Tham YJ (2014). Presence of high nitryl chloride in Asian coastal environment and its impact on atmospheric photochemistry. Chin. Sci. Bull..

[27] Oum KW, Lakin MJ, DeHaan DO, Brauers T, Finlayson-Pitts BJ (1998). Formation of molecular chlorine from the photolysis of ozone and aqueous sea-salt particles. Science.

[28] Knipping EM (2000). Experiments and simulations of ion-enhanced interfacial chemistry on aqueous NaCl aerosols. Science.

[29] Hoffmann EH, Tilgner A, Wolke R, Herrmann H (2019). Enhanced chlorine and bromine atom activation by hydrolysis of halogen nitrates from marine aerosols at polluted coastal areas. Environ. Sci. Technol..

[30] Yang X (2018). Abundance and origin of fine particulate chloride in continental China. Sci. Total Environ..

[31] George IJ, Anastasio C (2007). Release of gaseous bromine from the photolysis of nitrate and hydrogen peroxide in simulated sea-salt solutions. Atmos. Environ..

[32] Abbatt J (2010). Release of gas-phase halogens by photolytic generation of OH in frozen halide−nitrate solutions: an active halogen formation mechanism?. J. Phys. Chem. A.

[33] Richards NK, Finlayson-Pitts BJ (2012). Production of gas phase NO_2_ and halogens from the photochemical oxidation of aqueous mixtures of sea salt and nitrate ions at room temperature. Environ. Sci. Technol..

[34] Mack J, Bolton JR (1999). Photochemistry of nitrite and nitrate in aqueous solution: a review. J. Photochemistry Photobiol. A: Chem..

[35] Benedict KB, McFall AS, Anastasio C (2017). Quantum yield of nitrite from the photolysis of aqueous nitrate above 300 nm. Environ. Sci. Technol..

[36] Scharko NK, Berke AE, Raff JD (2014). Release of nitrous acid and nitrogen dioxide from nitrate photolysis in acidic aqueous solutions. Environ. Sci. Technol..

[37] Zellner R, Exner M, Herrmann H (1990). Absolute OH quantum yields in the laser photolysis of nitrate, nitrite and dissolved H_2_O_2_ at 308 and 351 nm in the temperature range 278–353 K. J. Atmos. Chem..

[38] Jayson GG, Parsons BJ, Swallow AJ (1973). Some simple, highly reactive, inorganic chlorine derivatives in aqueous solution. Their formation using pulses of radiation and their role in the mechanism of the Fricke dosimeter. J. Chem. Soc., Faraday Trans. 1: Phys. Chem. Condens. Phases.

[39] Ye C, Zhang N, Gao H, Zhou X (2017). Photolysis of particulate nitrate as a source of HONO and NOx. Environ. Sci. Technol..

[40] Zhao J, Zhang Y, Quan X, Chen S (2010). Enhanced oxidation of 4-chlorophenol using sulfate radicals generated from zero-valent iron and peroxydisulfate at ambient temperature. Sep. Purif. Technol..

[41] Wall KJ, Harris GW (2017). Uptake of nitrogen dioxide (NO_2_) on acidic aqueous humic acid (HA) solutions as a missing daytime nitrous acid (HONO) surface source. J. Atmos. Chem..

[42] Al-Obaidi U, Moodie RB (1985). The nitrous acid-catalysed nitration of phenol. J. Chem. Soc., Perkin Trans..

[43] Richards-Henderson NK (2013). Production of gas phase NO_2_ and halogens from the photolysis of thin water films containing nitrate, chloride and bromide ions at room temperature. Phys. Chem. Chem. Phys..

[44] Edwards PM (2014). High winter ozone pollution from carbonyl photolysis in an oil and gas basin. Nature.

[45] Pye HO (2020). The acidity of atmospheric particles and clouds. Atmos. Chem. Phys..

[46] Fu X (2020). Persistent heavy winter nitrate pollution driven by increased photochemical oxidants in northern China. Environ. Sci. Technol..

[47] Bian H (2017). Investigation of global particulate nitrate from the AeroCom phase III experiment. Atmos. Chem. Phys..

[48] Fu X (2018). Anthropogenic emissions of hydrogen chloride and fine particulate chloride in China. Environ. Sci. Technol..

[49] McNamara SM (2020). Observation of road salt aerosol driving inland wintertime atmospheric chlorine chemistry. ACS Cent. Sci..

[50] Willis MD, Leaitch WR, Abbatt JPD (2018). Processes controlling the composition and abundance of arctic aerosol. Rev. Geophysics.

[51] Sharma S (2019). A factor and trends analysis of multidecadal lower tropospheric observations of arctic aerosol composition, black carbon, ozone, and mercury at Alert, Canada. J. Geophys. Res.: Atmospheres.

[52] Roberts JM, Osthoff HD, Brown SS, Ravishankara AR (2008). N_2_O_5_ oxidizes chloride to Cl_2_ in acidic atmospheric aerosol. Science.

[53] Wexler AS, Clegg SL (2002). Atmospheric aerosol models for systems including the ions H^+^, NH_4_^+^, Na^+^, SO_4_^2−^, NO_3_^−^, Cl^−^, Br^−^, and H_2_O. J. Geophys. Res.: Atmospheres.

[54] Tan Z (2017). Radical chemistry at a rural site (Wangdu) in the North China Plain: observation and model calculations of OH, HO_2_ and RO_2_ radicals. Atmos. Chem. Phys..

[55] Wang X, Wang H, Wang S (2010). Ambient formaldehyde and its contributing factor to ozone and OH radical in a rural area. Atmos. Environ..

[56] Trebs I (2009). Relationship between the NO_2_ photolysis frequency and the solar global irradiance. Atmos. Meas. Tech..

[57] Xue L (2015). Development of a chlorine chemistry module for the Master Chemical Mechanism. Geoscientific model Dev..

[58] Ammann M (2013). Evaluated kinetic and photochemical data for atmospheric chemistry: Volume VI – heterogeneous reactions with liquid substrates. Atmos. Chem. Phys..

