# Supplementary material for: Photodissociation of particulate nitrate as a source of daytime tropospheric Cl2
Source: Nat Commun. 2022 Feb 17;13:939. doi: 10.1038/s41467-022-28383-9 (PMC8854671; doi:10.1038/s41467-022-28383-9)
Supplement: Supplementary file 1 — Supplementary Information [file 41467_2022_28383_MOESM1_ESM.pdf]

Supplementary Information for

Photodissociation of particulate nitrate as a source of daytime tropospheric Cl_2

Xiang Peng[1,2], Tao Wang[1*,] Weihao Wang[1,3], A.R. Ravishankara[4], Christian George[5], Men Xia[1], Min Cai[6], Qinyi Li[7], Christian Mark Salvador[8,15], Chiho Lau[9], Xiaopu Lyu[1], Chun Nan Poon[1], Abdelwahid Mellouki[6], Yujing Mu[10], Mattias Hallquist[8], Alfonso Saiz-Lopez[7], Hai Guo[1], Hartmut Herrmann[11,12], Chuan Yu[1,13], Jianing Dai[1,16], Yanan Wang[1], Xinke Wang[5], Alfred Yu[9], Kenneth Leung[9], Shuncheng Lee[1], and Jianmin Chen[14]

*email: cetwang@polyu.edu.hk

Contents:

Supplementary Figures: Supplementary Fig. 1 to Supplementary Fig. 14

Supplementary Tables: Supplementary Table 1 to Supplementary Table 3

References

Supplementary Figures:

Supplementary Fig. 1. The locations of the measurement site in Cape D'Aguilar (also called Hok Tsui) in Hong Kong (yellow star). (Map credit: Google Earth)

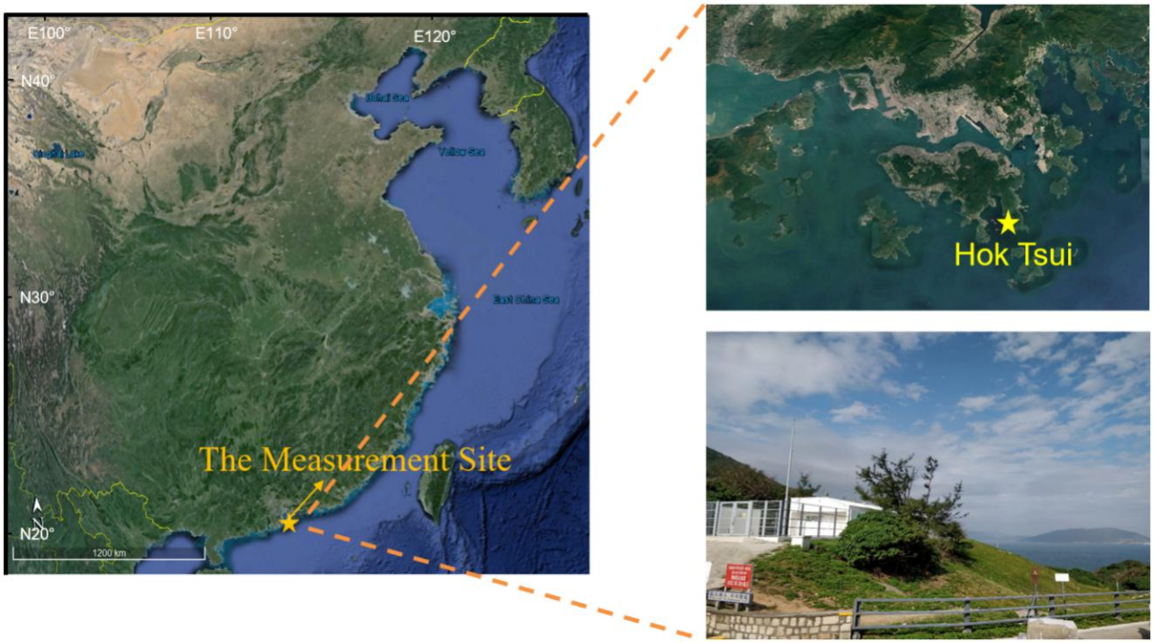

Supplementary Fig. 2. Ambient observations from 31 August to 9 October of 2018 in the clean air mass which originated from the ocean and in the polluted air mass which originated from the continental region. The measurements during 14-21 September were interrupted due to a super typhoon (Mangkhut) hitting the south China coast (including Hong Kong).

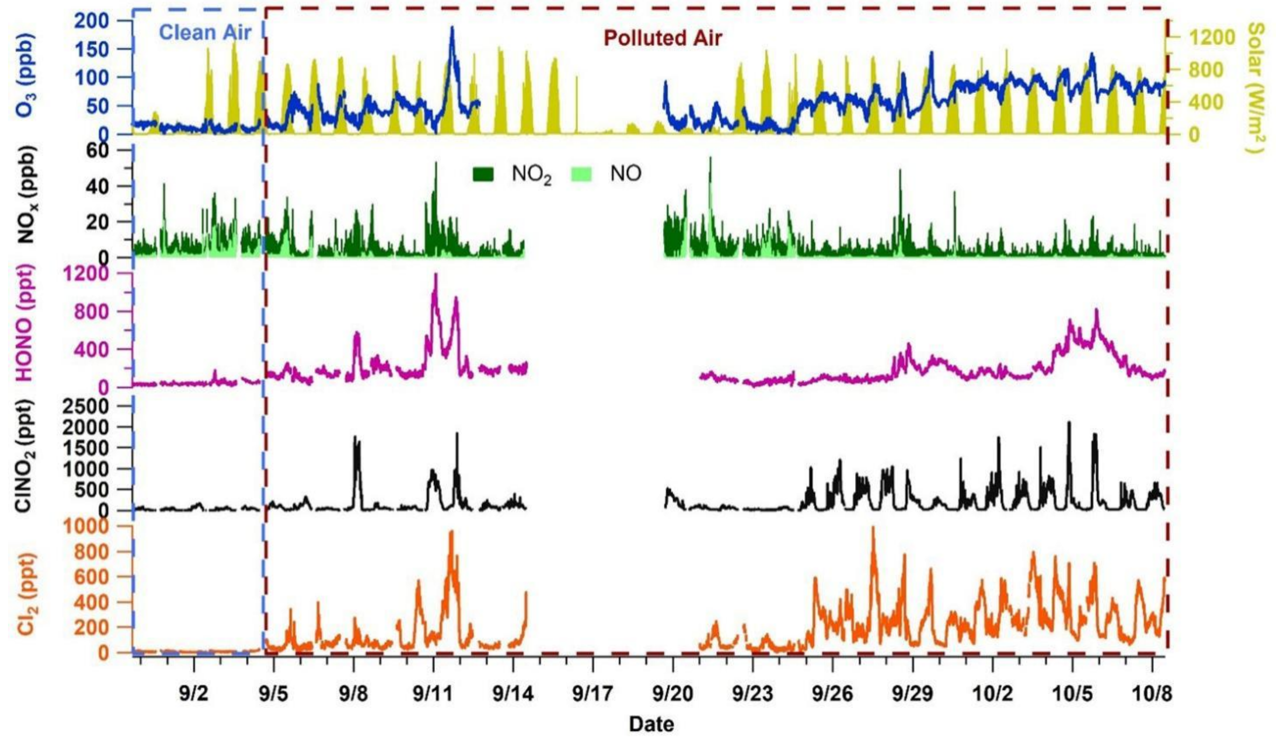

Supplementary Fig. 3. The model predicted average diurnal profiles of HOCl and $ClONO_2$ averaged for the period of 4 -14 September 2018.

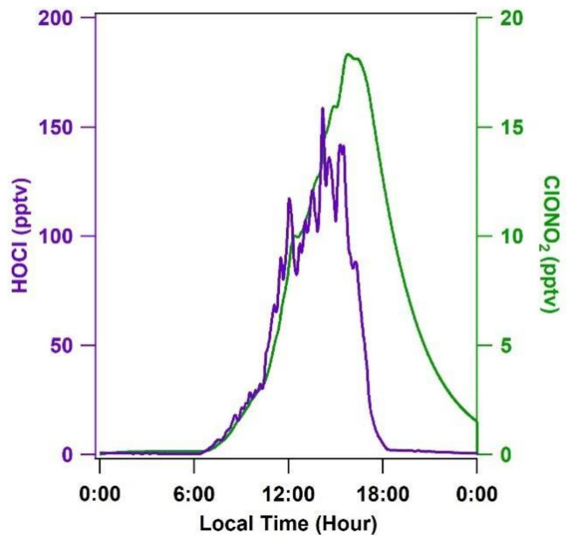

Supplementary Fig. 4. Average diurnal profiles of select input parameters used in the model simulation (4-14 September 2018).

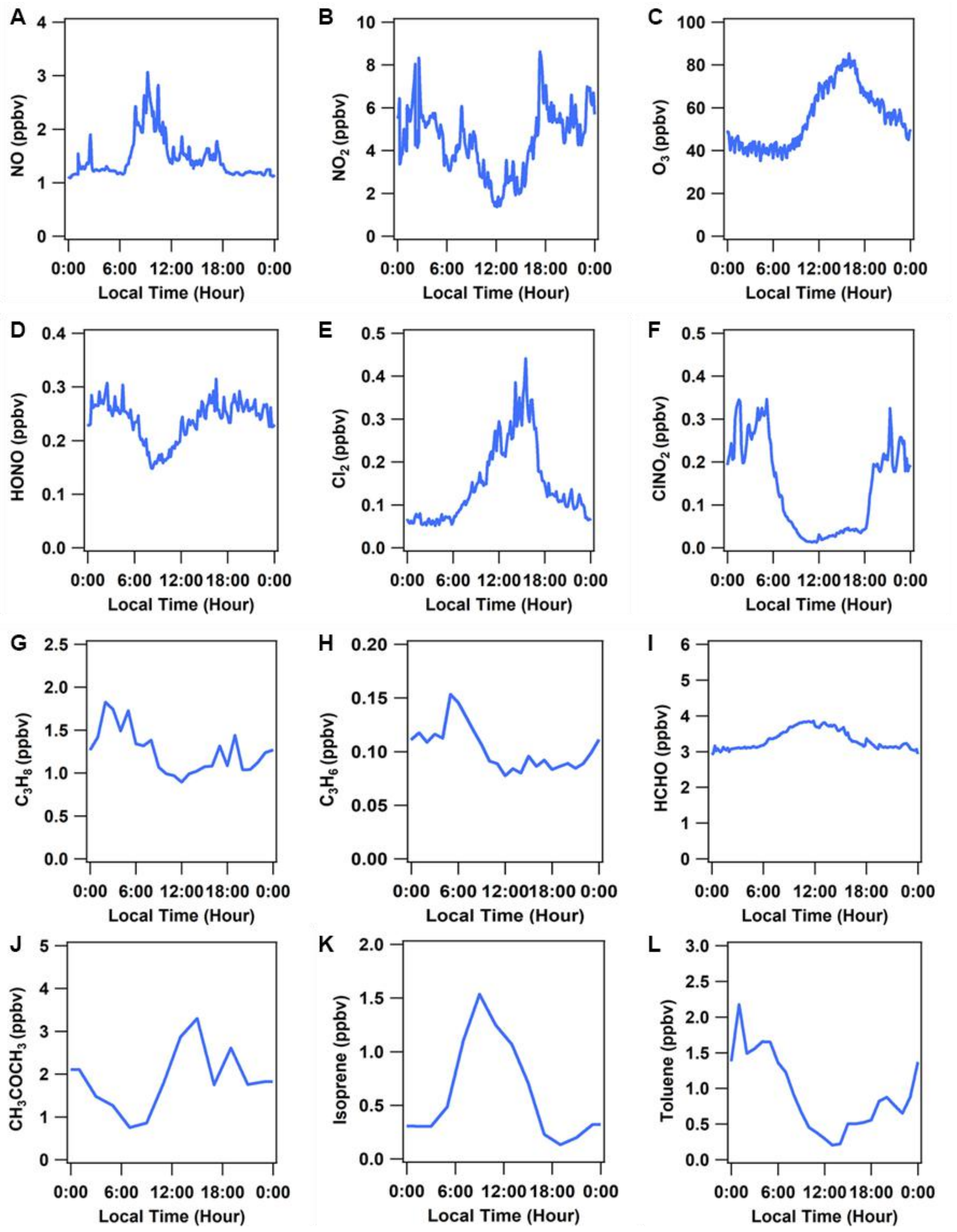

Supplementary Fig. 5. Scatter plot of the production rate of Cl_2 (P_{Cl2}) and various measured parameters from 08:00 to 18:00 in the continental air mass during 5 September and 9 October 2018. The P_{Cl2} equals the photolysis rate of Cl_2 (J_{Cl2} × measured Cl_2 concentration), assuming Cl_2 in a photo stationary state (given its short lifetime of ~7 minutes at noob in our study). J_{Cl2} was calculated from the TUV model (http://cprm.acom.ucar.edu/Models/TUV/Interactive_TUV) under clear sky conditions and then scaled to the solar irradiation derived J_{NO2} (see Methods section 3). The Sa represents the aerosol surface area density (μm^2 cm^{-3}).

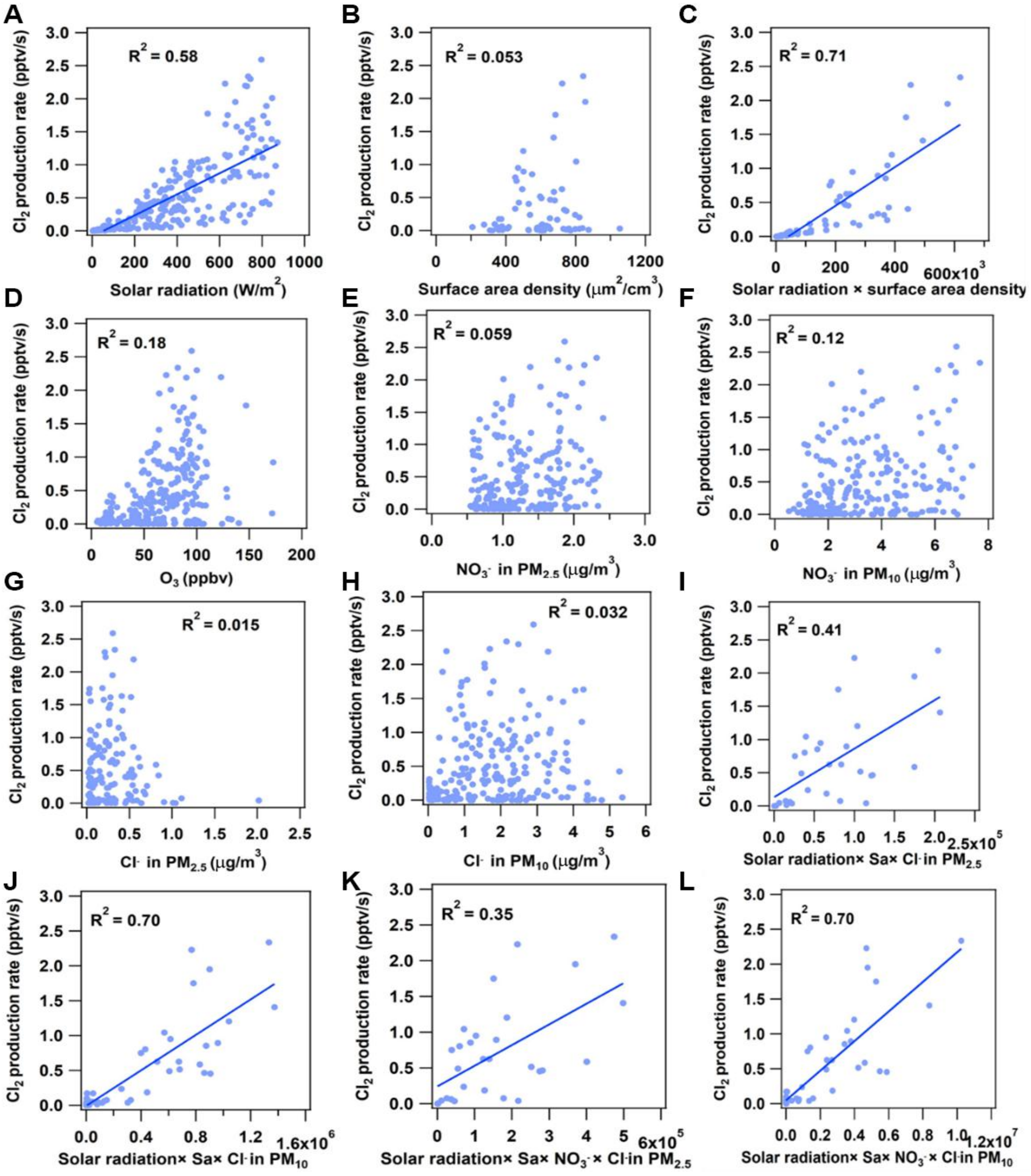

Supplementary Fig. 6. The irradiation spectrum of the xenon lamp used in this study and the Cl_2 cross-section (IUPAC) (http://iupac.pole-ether.fr/index.html).

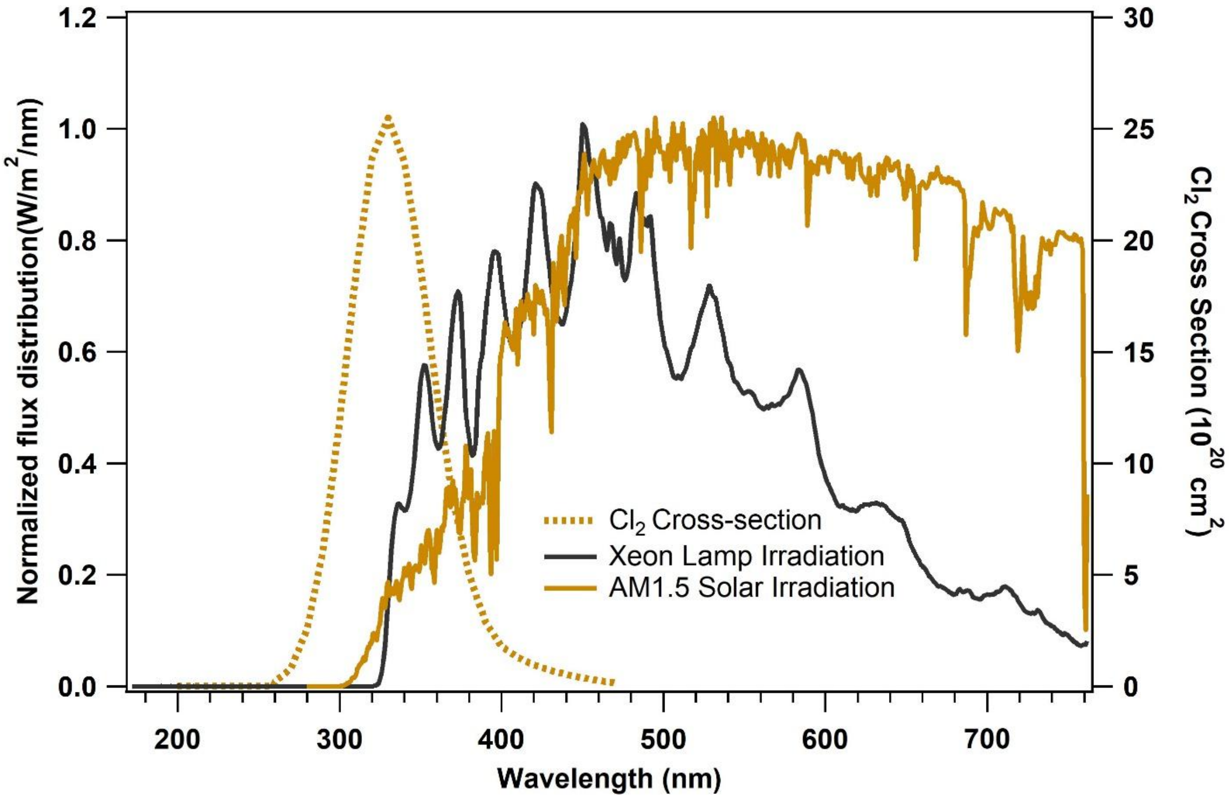

Supplementary Fig. 7. The schematic and photos of the experimental apparatus for Cl_2 production by irradiation. The chamber is made of TFE Teflon (1.875L, 25cm-length × 15cm-width × 4cm-height) with a Teflon-film window on the top.

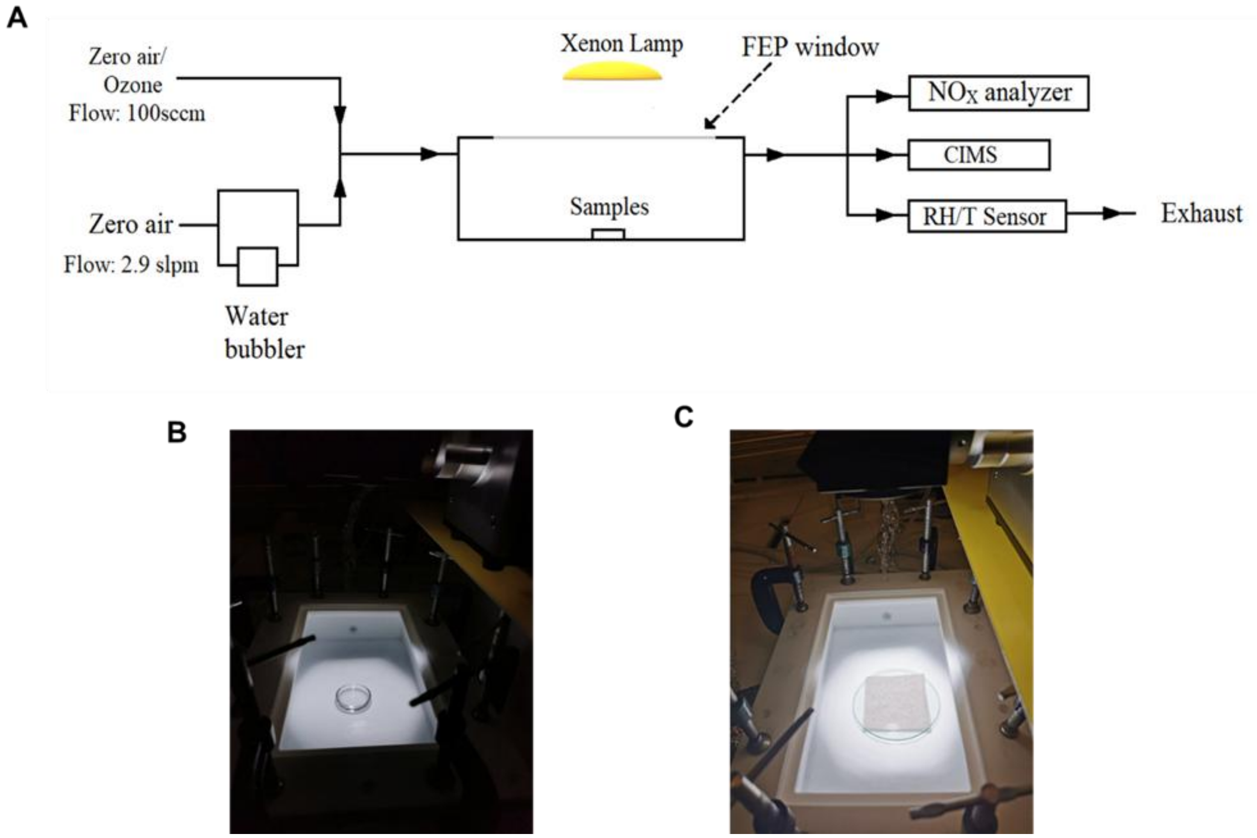

Supplementary Fig. 8. Ozone experiment results on solutions. (**A**) Comparison of 1-min average Cl_2 mixing ratios without and with ozone. Acidic liquid solution samples (pH=1.9) were illuminated at t=0. The green line represents the result without ozone, and the orange cycle represents result with ozone. In the ozone test, about 500 ppbv ozone was added at t=- 60 min. (**B**) Time series of Cl_2 mixing ratios with the addition of various levels of ozone. Liquid solution samples (pH=3.9) were illuminated at t=0. About 150 ppbv ozone was added at t=- 60 min., and the ozone level was changed to 250 ppbv at t=120, and further changed to 500 ppbv at t=180 min. The xenon lamp was turned off at t=240 min. Experimental conditions: 75-83% RH, 298 K in air, 15A Xenon Lamp, and one 4 ml liquid solution sample containing 1M NaCl + 1M $NaNO_3$.

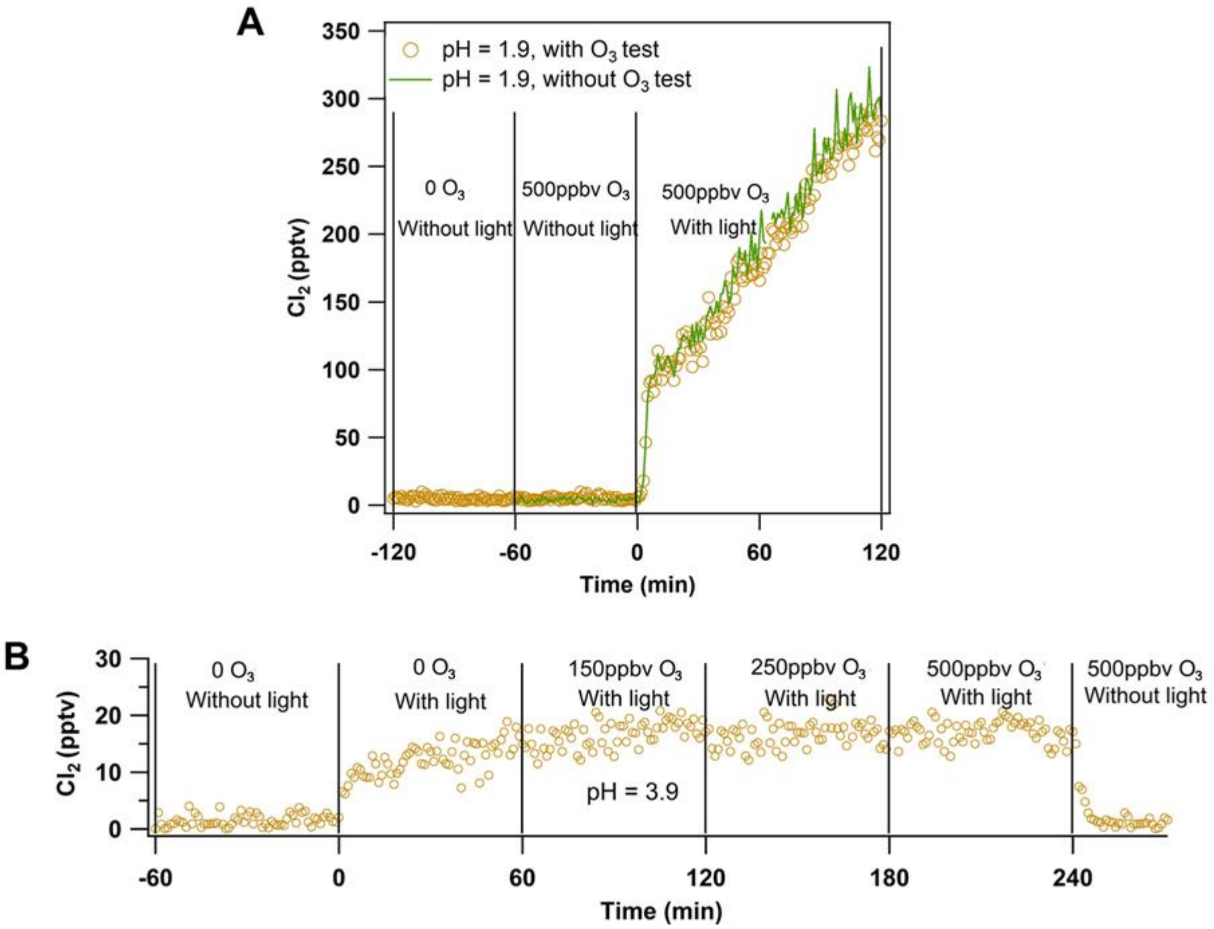

Supplementary Fig. 9. Experimental results on solutions with different initial pH. Time series of 1-min average Cl_2. Liquid solution samples (with the initial pH of 1.9, 2.9, and 6.8) were illuminated at t=0. The left insert: dependence of the Cl_2 yield (the production of Cl_2) as a function of time under the initial pH= 1.9. The orange cycle represents the use of one petri dish with 4ml solution, and the orange line represents the use of four Petri dishes with 1ml solution per petri dish. The right insert: the enlarged experimental results on solutions with the initial pH of 6.8. Experimental conditions: 75-83% RH, 298 K in air, 15A xenon lamp, and total 4 ml liquid solution containing 1M NaCl + 1M $NaNO_3$.

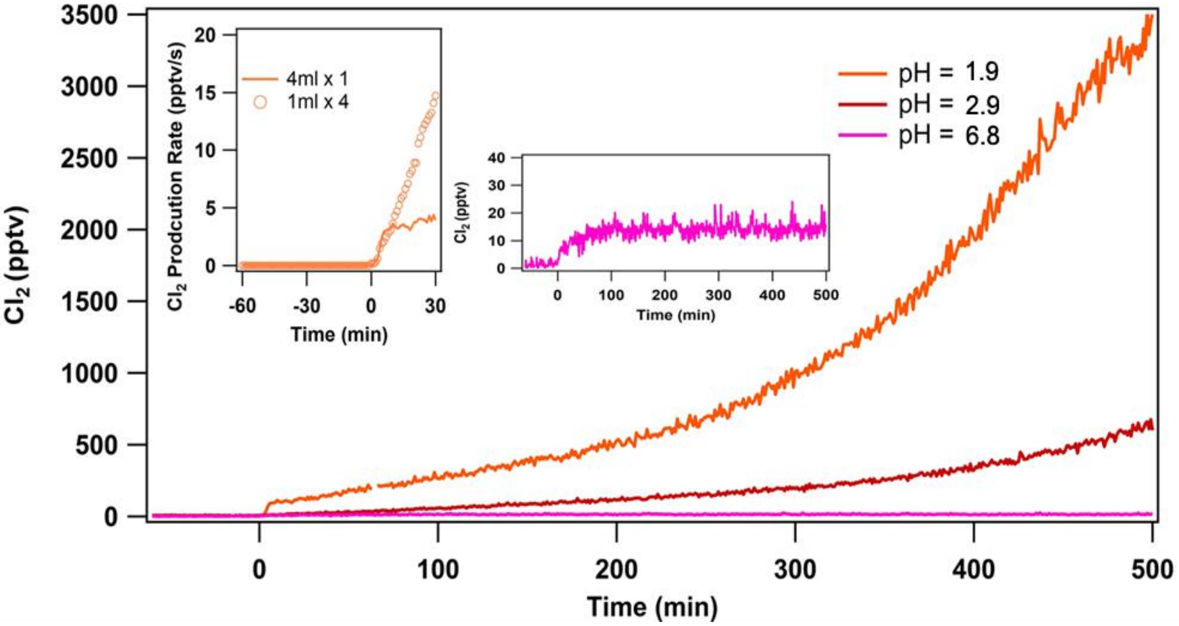

Supplementary Fig. 10. Relative frequency distribution of E-AIM calculated pH of hourly aerosol in $PM_{2.5}$ during the Hok Tsui observation from 31 August to 9 October in 2018. The number of data points is 555. The red dashed line represents a regression of the pH by Gaussian distributions. Details of E-AIM model setup are as follows. Model III with the batch mode was selected. The default temperature, pressure, and volume were adopted as 298.15 K, 1 atm, and 1 m^3, respectively. H^+ was set to balance the charges of anions and cations. Br^- and OH^- were set as zero. Water dissociation is considered (parameter e=1). Gas-phase HNO_3, HCl, NH_3, and H_2SO_4 are allowed and are partitioned between the gas phase and the condensed phases (parameter p, q, r, s = 0). The model is configured to search all the possible solids (parameter u=0). Organic compounds are not considered in the model. The E-AIM estimated average equivalent Cl^- molarity in $PM_{2.5}$ was 0.10 mol L^{-1} (standard deviation: 0.19 mol L^{-1}) and gas-phase HCl concentration was 0.96 ppbv (standard deviation: 0.52 ppbv).

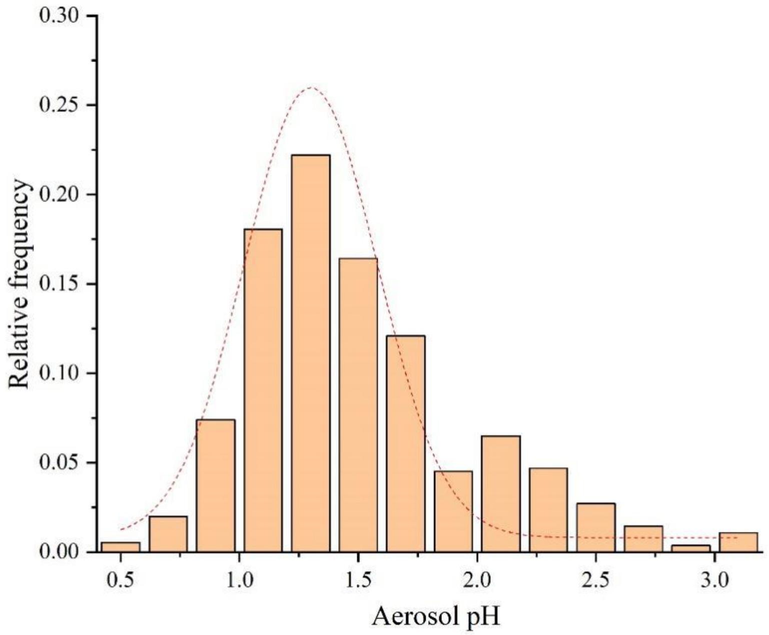

Supplementary Fig. 11. The model calculated contributions of net ozone production rates and radical abundance averaged for the period of 4 -14 September 2018. (**A**) The average diurnal profiles of Cl atom concentrations. (**B**) The average diurnal profiles of the net production rate of O_x (= O_3 + NO_2) (different color bars). The blue bar and black bar represent results without Cl chemistry and with Cl chemistry, respectively. The red line represents field measurements of O_x. (**C**) The average diurnal profiles of OH, HO_2, and RO_2. The blue and black bars have the same meaning as panel (B).

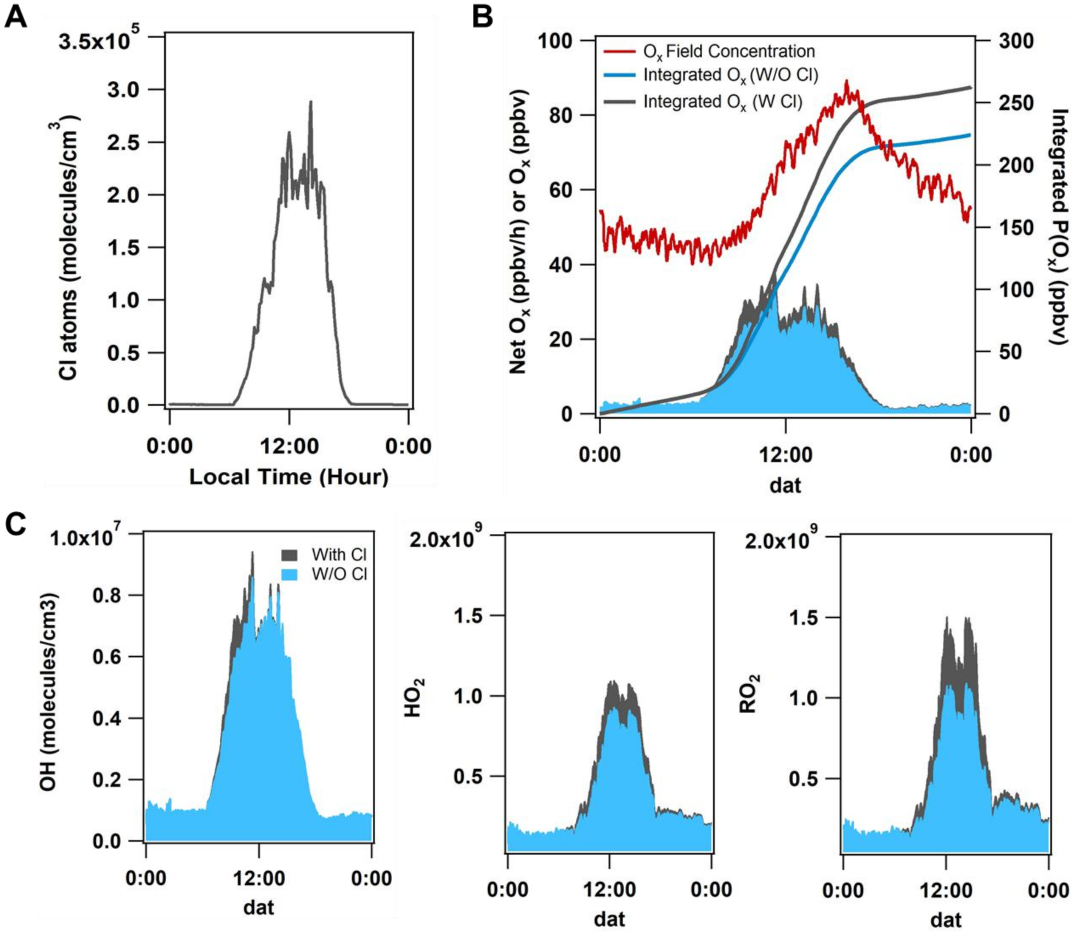

Supplementary Fig. 12. Calculated hydrocarbon oxidation rates by different oxidants. Relative contributions to the daily integrated oxidation of alkanes, alkenes (without dialkenes), aromatics, alcohols aldehyde, ketones, and dialkenes by OH, Cl, NO_3, and O_3 (averaged for the time period of 4-14 September of 2018).

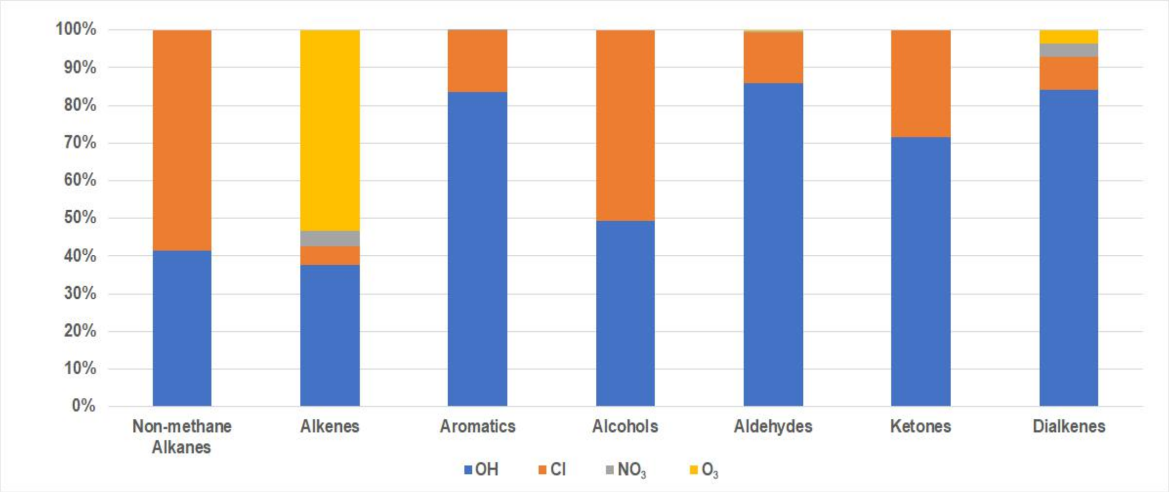

Supplementary Fig. 13. Mean diurnal profile of Cl_2 and CMBO. Besides the inorganic chloride species, organochlorides (ClOVOCs) were also measured at the same measurement site in Hong Kong using the High-Resolution Time of Flight Chemical Ionization Mass Spectrometer (HR-ToF-CIMS) but at different period (14-26 November 2018). The detailed information of the instrument and identification of Cl-VOCs can be found in the previous study [1]. Briefly, the HR-ToF-CIMS adopts chemical reactions to ionize the target gases using iodide (I^-) as the reagent ion. Along with Cl_2, thirteen gas-phase C_1-C_6 ClOVOCs were detected, with 1-chloro-3-methyl-3-butene-2-one (CMBO, C_5H_6ClO) as the most dominant organochloride. Cl_2 and CMBO were detected as iodide adducts (ICl_2^- and $IC_5H_6ClO^-$, respectively) after ion-molecule reactions: $I^- + Cl_2 \rightarrow ICl_2^-$, $I^- + C_5H_6ClO \rightarrow IC_5H_6ClO^-$. Other species were measured with the similar ionization chemistry. CMBO is the chlorine oxidation product of isoprene, which makes this ClOVOC a unique tracer of chlorine-biogenic chemistry [2,3]. The daily maxima of CMBO coincided with that of Cl_2, indicative of VOC oxidation by Cl atom. The color region represents the standard deviation of the data set. This will be explained further in succeeding studies. No calibration was conducted, and therefore the Cl_2 and CMBO measurements shown here are in arbitrary units.

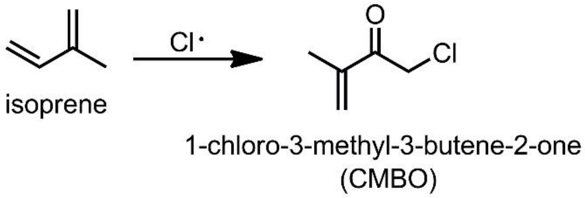

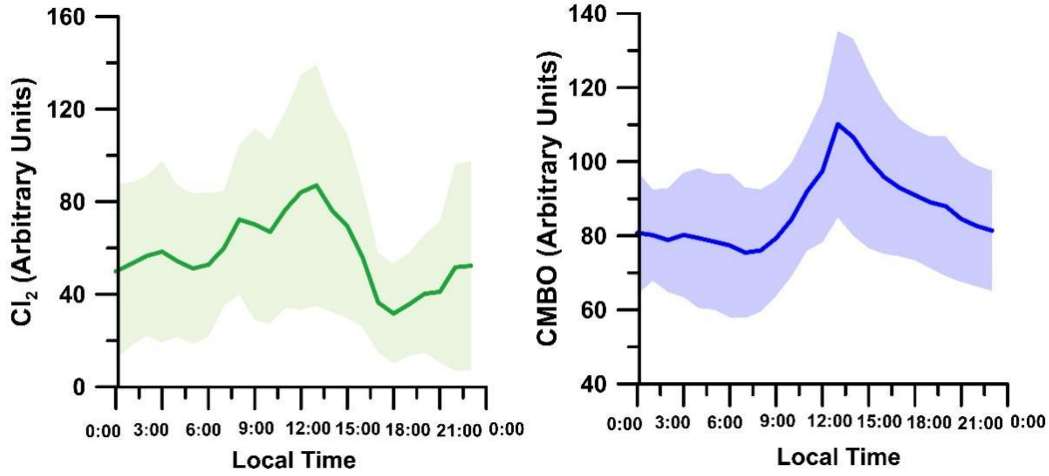

Supplementary Fig. 14. The CIMS performance for Cl_2 ambient measurement from 31 August to 9 October of 2018. (**A**) An example of the mass spectrum of CIMS from 120 amu to 220 amu during the field measurements. The signals below 10 Hz were not recorded during hourly scans but were recorded during measurements. The insert panels are the high-resolution scan spectra for Cl_2. (**B**) The background level of Cl_2 (the signal equivalent to concentration) during the campaign. (**C**) Scatter plot of the raw CIMS signal of Cl_2 at mass 199 amu ($I^{35}Cl^{37}Cl^-$; $I^{37}Cl^{35}Cl^-$)) versus 197 amu ($I^{35}Cl^{35}Cl^-$) with 10 min average for the entire ambient measurement period. The blue lines are the measured ratios, and the red dashed lines are the theoretical isotopic ratios. (**D**) The sensitivity of Cl_2 was determined on-site to confirm the stability of CIMS. (**E**)The sensitivity of Cl_2 under different RH in dilution zero air.

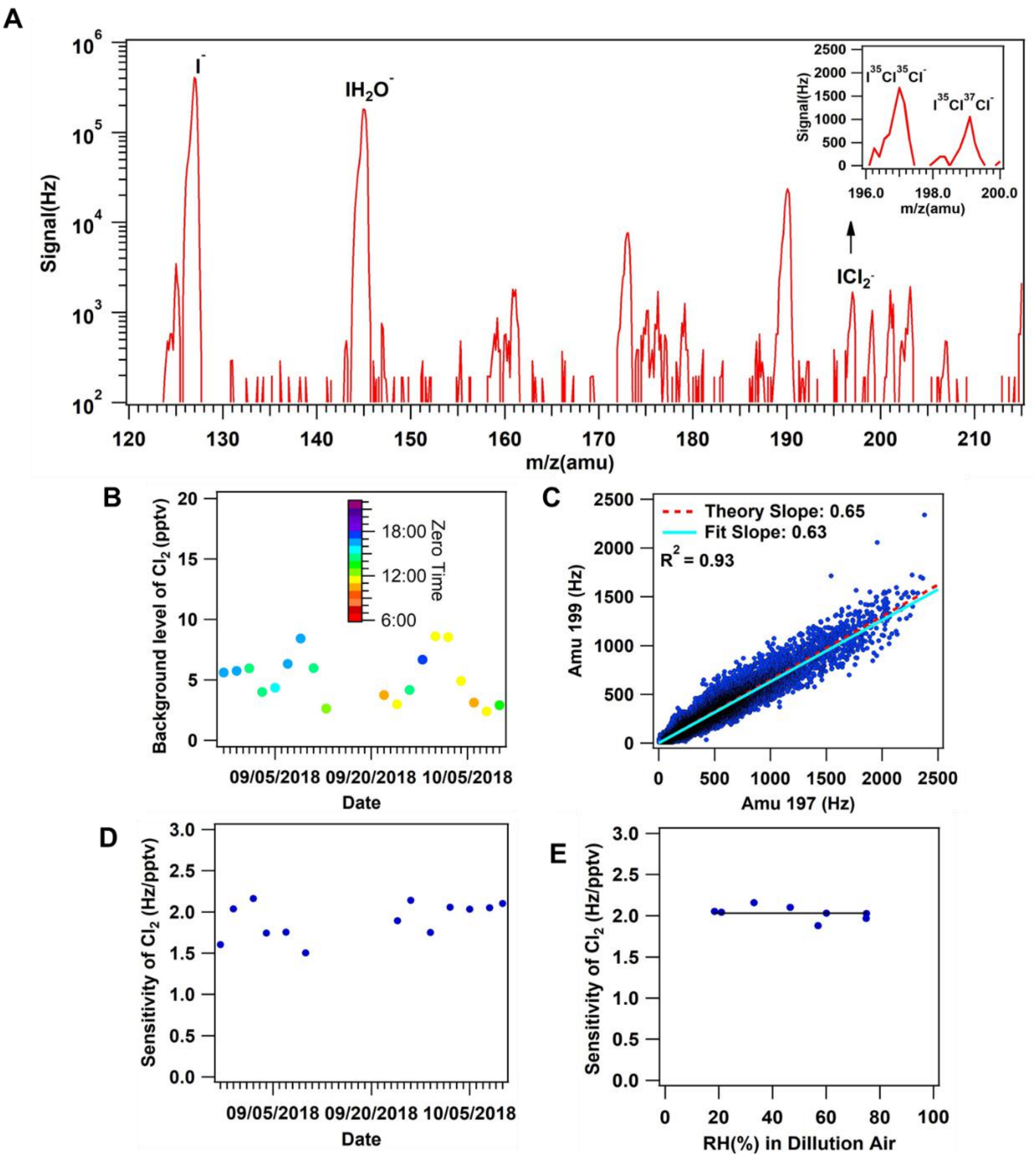

Supplementary Tables:

Supplementary Table 1. Input parameters to the box model for halogen impact evaluation. All listed parameters (except for CH_4 and HCHO) in the table were the concurrent measurement data at our site for period 4-14 September 2018. The VOCs names are given in MCM format.

No	Parameter	Time resolution	Average value ± Standard deviation
1	Temperature	1 min	27.7±1.25 °C
2	RH	1 min	82.8±4.42%
3	JNO2	1 min	0.0021±0.0026 s^{-1}
4	NO	1 min	1.46±0.385 ppbv
5	NO2	1 min	4.45±1.53 ppbv
6	O3	1 min	55.4±14.0 ppbv
7	CO	1 min	260±10.2 ppbv
8	SO2	1 min	1.77±0.367 ppbv
9	N2O5	1 min	0.051±0.051 ppbv
10	ClNO2	1 min	0.139±0.106 ppbv
11	Cl2	1 min	0.149±0.091 ppbv
12	HONO	1 min	0.238±0.0373 ppbv
13	C2H6	1 min	1.21±0.209 ppbv
14	C2H4	1 min	0.208±0.0472 ppbv
15	C3H8	1 min	1.25±0.242 ppbv
16	C3H6	1 min	0.102±0.0196 ppbv
17	IC4H10	1 min	0.842±0.288 ppbv
18	NC4H10	1 min	1.35±0.648 ppbv
19	TBUT2ENE	1 min	0.627±0.153 ppbv
20	BUT1ENE	1 min	0.0530±0.0106 ppbv
21	IC5H12	1 min	0.529±0.130 ppbv
22	NC5H12	1 min	0.448±0.065 ppbv
23	BENZENE	1 min	0.270±0.145 ppbv
25	TOLUENE	1 min	0.905±0.511 ppbv
26	CH3CHO	1 min	1.141±0.865 ppbv
27	Cyclopentane	1 min	0.100±0.0284 ppbv
28	Methylcyclopentane	1 min	0.169±0.0509 ppbv
29	2,2,4-Trimethylpentane	1 min	0.0567±0.0134 ppbv
30	C5H8	1 min	0.636±0.444 ppbv
31	C2H5CHO	1 min	0.289±0.0759 ppbv
32	CH3COCH3	1 min	1.86±0.667 ppbv
33	M22C4	1 min	0.0761±0.0194 ppbv
34	M2PE	1 min	0.199±0.0694 ppbv

35	NC6H14	1 min	0.841±0.568 ppbv
36	C3H7CHO	1 min	1.283±0.364 ppbv
37	M2HEX	1 min	0.0485±0.0261 ppbv
38	CHEX	1 min	0.143±0.0593 ppbv
39	M3HEX	1 min	0.260±0.0449 ppbv
40	NC7H16	1 min	0.110±0.0413 ppbv
41	C5H11CHO	1 min	0.148±0.0210 ppbv
42	C54CHO	1 min	0.142±0.0296 ppbv
43	EBENZ	1 min	0.242±0.125 ppbv
44	PXYL	1 min	0.514±0.329 ppbv
45	OXYL	1 min	0.227±0.160 ppbv
46	BENZAL	1 min	0.0639±0.0075 ppbv
47	MXYLAL	1 min	1.35±0.80 ppbv
48	CH4	1 min	2000±0 ppbv
49	HCHO	1 min	3.34±0.275 ppbv

Supplementary Table 2. The peak Cl_2 mixing ratios observed during illumination of four ambient filter samples and corresponding aerosol composition.

Filter Number	Cl_2 Concentration	Cl^- (μg m^{-3}) in Filter	NO_3^- (μg m^{-3}) in Filter
01	300 pptv	8.66	5.95
02	550 pptv	10.59	2.90
03	Below detection limit	2.30	1.05
04	Below detection limit	0.57	0.72

Supplementary Table 3. Instruments used in the field study.

Measured Species	Instrumentation	Time Resolution
Cl_2, $ClNO_2$, N_2O_5	Q-CIMS	1 min
* HONO	Q-CIMS	1 min
	LOPAP (QUMA, Model LOPAP-03)	10 min
NO, NO_2	Chemiluminescence/photolytic converter (Thermo, Model 42i)	1 min
O_3	UV photometric analyzer (Thermo, Model 49i)	1 min
** Compositions in $PM_{2.5}$ and PM_{10} (including NO_3^-, Cl^- $NH4^+$, SO_4^{2-})	MARGA	1 hour
Solar Radiation	Pyranometer (li-200, licor)	1 min
*** Dry-state particle number size distribution	WPS (model 1000XP, MSP Corporation)	
VOCs	GC-MS/FID (GC955 Series 611/811, Syntech Spectras)	1 hour
	off-line DNPH-Cartridge-HPLC	2 hours
	PTR-MS (PTR-QMS 500, IONICON Analytik, Austria)	10 min
OVOCs	off-line DNPH-Cartridge-HPLC	2 hours

* HONO was measured by CIMS and LOPAP in this study. The two instruments showed good agreement. The HONO data from the CIMS were used in model calculations.

** The molar concentrations of inorganic ions (i.e., [Cl^-], [NO_3^-], and [H^+]) in aerosol water were estimated using the extended aerosol inorganics model (E-AIM, model III) [4,5] (please see Methods section 2).

*** The dry-state particle number size distribution was measured by the WSP with a diffusion dryer, covering the size ranging from 10 nm to 10000 nm. The ambient (wet) particle number size distributions were calculated based on a size-resolved kappa-Köhler dependence on the relative humidity [6-9]. The aerosol surface area density was calculated

with the wet ambient particle number size distribution assuming spherical particles. In the present study, data with RH greater than 90 % were excluded due to the large uncertainty of the growth factor at very high RH.

Supplementary References

1. Le Breton, M. *et al.* Chlorine oxidation of VOCs at a semi-rural site in Beijing: significant chlorine liberation from ClNO2 and subsequent gas- and particle-phase Cl–VOC production. *Atmospheric Chemistry Physics* **18**, 13013-13030, doi:10.5194/acp-18-13013-2018 (2018).
2. Nordmeyer, T. *et al.* Unique products of the reaction of isoprene with atomic chlorine: Potential markers of chlorine atom chemistry. *Geophysical Research Letters* **24**, 1615-1618, doi:https://doi.org/10.1029/97GL01547 (1997).
3. Tanaka, P. L. *et al.* Direct evidence for chlorine-enhanced urban ozone formation in Houston, Texas. *Atmospheric Environment* **37**, 1393-1400, doi:https://doi.org/10.1016/S1352-2310(02)01007-5 (2003).
4. Wexler, A. S. & Clegg, S. L. Atmospheric aerosol models for systems including the ions H+, NH4+, Na+, SO42−, NO3−, Cl−, Br−, and H2O. *Journal of Geophysical Research: Atmospheres* **107**, ACH 14-11-ACH 14-14 (2002).
5. Xia, M. *et al.* Significant production of ClNO2 and possible source of Cl2 from N2O5 uptake at a suburban site in eastern China. *Atmospheric Chemistry Physics* **20**, 6147-6158 (2020).
6. Liu, H. J. *et al.* Aerosol hygroscopicity derived from size-segregated chemical composition and its parameterization in the North China Plain. *Atmospheric Chemistry and Physics* **14**, 2525-2539, doi:10.5194/acp-14-2525-2014 (2014).
7. Hennig, T., Massling, A., Brechtel, F. J. & Wiedensohler, A. A Tandem DMA for highly temperature-stabilized hygroscopic particle growth measurements between 90% and 98% relative humidity. *Journal of Aerosol Science* **36**, 1210-1223, doi:10.1016/j.jaerosci.2005.01.005 (2005).
8. Yu, C. *et al.* Heterogeneous N2O5 reactions on atmospheric aerosols at four Chinese sites: improving model representation of uptake parameters. *Atmospheric Chemistry and Physics* **20**, 4367-4378, doi:10.5194/acp-20-4367-2020 (2020).
9. Yun, H. *et al.* Nitrate formation from heterogeneous uptake of dinitrogen pentoxide during a severe winter haze in southern China. *Atmospheric Chemistry and Physics* **18**, 17515-17527, doi:10.5194/acp-18-17515-2018 (2018).
